# Review of Health Effects of Automotive Brake and Tyre Wear Particles

**DOI:** 10.3390/toxics13040301

**Published:** 2025-04-11

**Authors:** Athanasia Christou, Barouch Giechaskiel, Ulf Olofsson, Theodoros Grigoratos

**Affiliations:** 1 Joint Research Centre (JRC), European Commission, 21027 Ispra, Italy; achristo@kth.se; 2 Department of Machinedesign, KTH, Royal Institute of Technlogy, 11428 Stockholm, Sweden; ulfo@md.kth.se; 3 Directorate-General for Environment (DG-ENV), European Commission, 1040 Brussels, Belgium; theodoros.grigoratos@ec.europa.eu

**Keywords:** non-exhaust particles, tyre abrasion, brakes, microplastics, adverse health effect

## Abstract

Non-exhaust emissions from brakes and tyres are becoming the major transport-related contributor of particulate matter (PM) pollution in cities. Furthermore, tyre microplastics are the major contributor of unintentionally released microplastics in all environmental compartments. The European Union introduced for the first time worldwide limits for brakes (PM_10_) and tyres (total abrasion mass) with the Euro 7 regulatory step. Thus, the interest in brake and tyre particles regarding health and environmental impacts has significantly increased in recent years. In this review, we summarise studies that assessed the impact of brake and tyre particles on human, mammalian, aquatic, and terrestrial cells and organisms. Furthermore, we summarise the studies that compared the impact of brake and tyre particles to other sources. We also critically examine the sampling methodologies of brake and tyre particles for health and environmental impact studies.

## 1. Introduction

Air pollution emissions have declined in the last two decades as a result of regulatory action in different sectors. For example, between 2005 and 2022, the number of premature deaths in the European Union (EU) attributable to particulate matter (PM) fell by 45% [1]. Premature deaths are projected to further decrease by 65% by 2030. Despite this improvement, air pollution remains the largest environmental health risk in Europe. Exposure to PM above the World Health Organization (WHO) recommendations caused an estimated 239,000 premature deaths in 2022 in the EU. While poor air quality is a critical issue that affects the whole world, it impacts some places more than others. For instance, 70% of all premature deaths caused by poor air quality happen in Pacific Asia [2]. Besides health issues, air pollution increases healthcare costs, reduces life expectancy, and damages vegetation and ecosystems, water and soil quality, and local ecosystems.

Pollutants of increasing concern are microplastics (synthetic polymer particles below five millimetres that are organic and insoluble and resist degradation) [3,4]. Microplastics are often added to products such as the granular infill material used in artificial sport surfaces, cosmetics, medicines, and detergents; they are also used in packaging. Tyre wear contributes significantly to the unintentional release of microplastics into the environment [5]. The smaller ones are emitted into the atmosphere, while the majority (coarse size and larger) are deposited on the road or nearby areas [6,7,8]. Resuspension, wind, and rain remobilise them. The majority of the particles settle in the soil; however, some end up in water via drainage systems (with or without treatment). Environmental aging processes such as thermooxidation, photooxidation, ozonolysis, shear stress, biodegradation, and leaching then take place, altering the physical and chemical properties of microplastics [9]. Recent work has highlighted the atmosphere’s role in transporting microplastics to remote locations [10].

**Figure 1:**
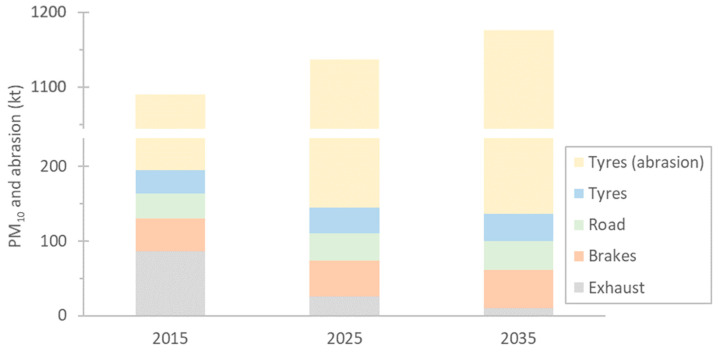
Particulate matter (PM_10_) from the road, vehicles’ exhaust, brakes, and tyres, and total wear from tyres. Estimations for the years 2015, 2025, and 2035 assuming no mitigation measures (based on [5,11]).

Figure 1 plots the estimated European Union 27 (EU27) emissions of PM_10_ and tyre microplastics into the environment for the years 2015, 2025, and 2035, assuming no mitigation actions [5,11]. Regarding PM, exhaust emissions will decrease significantly, leaving non-exhaust emissions as practically the main source of PM pollution. Electric vehicles still produce tyre particles, but they are expected to emit much fewer brake particles due to regenerative braking. However, it is always important to keep in mind that they do still produce brake wear particles, and corrosion over time that occurs as a result of fewer braking events might increase them. Brake particles have size distributions that peak at around 2–5 μm, which practically means most of the mass lies below 10 μm [11,12]. Tyre abrasion, which is in the order of 1000 kt per year, is the main source of non-exhaust microplastics [5]. In the EU, with Euro 7, worldwide non-exhaust emission limits were introduced for the first time. For brakes, PM_10_ will be regulated, while for tyres, the total abrasion will be regulated.

The contribution of non-exhaust sources to the total PM depends on the contribution of other sources as well, such as power generation plants, industry, the sea, and biomass burning [13,14], but also on road maintenance, weather conditions, and driving conditions. Resuspended dust, which includes previously emitted non-exhaust particles, can have a big impact on the absolute PM levels. As an example, PM_10_ levels can be significantly different in developed countries compared to countries where less attention is paid to the infrastructure [15]. While the exact composition of the tyres is an industry secret, they can be categorised into a few different groups. Summer tyres tend to be harder in nature in order to resist higher temperatures, while winter tyres tend to be softer in order to stay flexible in lower temperatures, and they usually have a larger, more complex tread pattern to support safe driving in wet conditions. Another commonly used tyre is the “all-season” tyre, which combines the technologies used in both summer and winter tyres in order to provide safe driving in all weather conditions. However, “all-season” tyres have caused some controversy, as it is believed that they provide more of a compromise between summer and winter tyres, not really being ideal for either season. Lastly, there are studded winter tyres which are used in many Nordic countries where the weather conditions are extreme. The hard metal studs installed in the tyre tread provide higher friction on slippery surfaces like icy roads. The general composition of tyre treads consists of rubber polymers (40–60%), reinforcing/filler agents (20–45%), and chemical additives (5–15%) [5]. Inorganic compounds such as Si, S, Zn, Ca, Al, and Fe and organic compounds such as benzothiazoles, N-(1,3-dimethylbutyl)-N′-phenyl-1,4-phenylenediamine (6PPD), and polycyclic aromatic hydrocarbons (PAHs) can be found in high concentrations. It is important to note that there is also a significant difference between light-duty and heavy-duty tyres and their composition. The particles found in the environment are tread particles encrusted and mixed with foreign materials from the road, dust, brakes, and soil.

The aim of a mechanical car brake system is to slow down or stop the vehicle by dissipating its kinetic energy and transforming it into frictional heat. In modern light-duty vehicles, two different kinds of brake systems are typically used: disc brake and drum brake systems. Drum brake systems consist of a spread mechanism that pushes two brake shoes against the inner surface of a cast iron or steel brake drum, connected to the wheel. A disc brake system consists of two main parts: the rotor and the pads. To allow a braking action to take place, the pads are pressed to the rotors. Because of their superior heat capacity, discs brakes are arguably safer than drum brakes on the front wheels.

The pad material differs depending on the region and its requirements. Non-asbestos organic (NAO) pads are commonly used in the US and Japanese market for comfort reasons. On the other hand, due to performance requirements, low-steel pads are common in Europe, together with semi-metallic ones, which have improved performance at high temperatures. This affects the chemical composition of the pad materials and potentially their toxic effects. To comply with the new Euro 7 regulations, hybrid pad materials that are a mix of NAO and low-steel pad ingredients have also been developed. Brake pads generally comprise five main components: binders (mainly phenol–formaldehyde resins), reinforcing fibres (copper, steel, brass, potassium titanate, glass, organic material (aramid), and Kevlar), fillers (barium and antimony sulphate, magnesium oxides and chromium oxides, silicates, ground slag, stone, and metal powders), frictional additives or lubricants, and abrasives (aluminium oxide, iron oxides, quartz, and zircon) [16,17,18]. Brake particles do not only originate from the pads but also from the disc which the brake pads are forced against. Discs used in passenger cars are typically made of grey cast iron, but in some cases, they can be made of reinforced carbon–carbon, ceramic matrix composites, or aluminium or even coated with materials that alter their properties and reduce wear.

Heavy-duty vehicles also use disc and drum brakes, in addition to different kinds of auxiliary brakes. Auxiliary brakes can be separated into engine brakes, electromagnetic auxiliary brakes, hydraulic auxiliary brakes, and compression release engine brakes. For the heavy-duty sector, the brake pad material composition differs for different market segments. For example, the bus segment prefers comfort and low noise emission, while long-distance trucks demand higher performance, i.e., higher coefficient of friction. New solutions are anticipated for the forthcoming Euro 7 regulation for the heavy-duty sector.

Many compounds from tyres and brakes have been found to be harmful, such as Zn, Cu, and PAHs [19,20,21,22]. Some tyre substances become more toxic when exposed to ultraviolet (UV) radiation. An example of such an oxidation product is 6PPD quinone [23]. There are a few reviews assessing the health and environmental impact of brake and tyre particles (e.g., [22,24] (and more will be presented in the next section)). However, a comprehensive review of the impact of both tyre and brake particles on mammalian, aquatic, and terrestrial organisms is missing.

Based on this background, the aim of this paper is to summarise the dedicated studies that have examined the impact of brake and tyre particles on health and the environment. Although there are reviews on this topic, studies have increased significantly in the last years. Furthermore, we try to understand whether non-exhaust particles are more or less harmful than other particles. Finally, we try to critically review the sampling methodologies in order to give recommendations for future studies.

## 2. Materials and Methods

### 2.1. Search Methodology

We searched articles in the PubMed and Google Scholar databases (accessed on 20 December 2024) using various keyword combinations, including “brakes”, “tyres”, “tires”, “non-exhaust”, ”particles”, “particulate matter”, “emissions” “health”, “human”, “aquatic”, and “terrestrial”.

Only original articles published in English and accessible through open access or institutional subscriptions were considered. The initial search yielded many articles, which were subsequently filtered by reading the titles and abstracts. In addition to research articles, we separately assessed reviews on the topic. Furthermore, relative papers cited in those studies were also checked. Table 1 summarises the key reviews on the topic. Reviews of microplastics are not included (e.g., [25,26,27,28,29,30]).

### 2.2. Methods of Toxicological Studies

In this paper, we looked at toxicological studies, as there were almost no epidemiological studies published on the health effects of non-exhaust emissions. The main reason for this lack of epidemiological studies is the ethical concerns around it. Long-term studies exposing humans to potentially harmful emissions, in order to study the effects, are not ethically correct as they would cause direct harm to the subjects involved. This is why most of the current studies are conducted on cells, mice, and other organisms. There are a few epidemiological studies looking at observational data; however, with such studies, it is often hard to establish a direct causal relationship between non-exhaust emissions and health outcomes, as humans are exposed to various different sources of pollution. This is why it is crucial to find certain markers that can point to these sources of pollution. Some studies indicated that some compounds typically found in brake particles (e.g., Cu) or tyres particles (e.g., Zn) are associated with adverse cardiovascular health outcomes or mortality [12,39,40,41]. On the other hand, one epidemiological study used barium as a marker for brake wear particles, which shows that markers do not necessarily prove the presence of a certain pollutant [42].

Another important observation is that a proportionally higher number of toxicological studies were conducted in vitro compared to in vivo. This happens because in vitro studies are much easier and cheaper to perform and have less limitations on exposure pathways and dosages. Studies on mammalian organisms were carried out using human or mice cells. On the other hand, most of the studies on aquatic or terrestrial organisms (not mammalian) were conducted in vivo.

Overall, most of the toxicological studies followed a similar methodology. For the in vitro studies, the exposure methods were either submersion or an air–liquid interface. On the other hand, in most in vivo studies, the exposure method was either inhalation or (intratracheal) installation. The benefit of intratracheal installation is that it directly deposits the substance in the airways, and it is much easier to control the exposure dosage. However, this method can often cause disturbances and trauma to the subject, which could, in turn, affect the results of the study [43,44,45].

The main parameters used to assess the toxicity of wear particles were the following:

- Reactive oxygen species (ROS), which are chemically reactive molecules containing oxygen often generated in response to environmental stressors.
- Interleukine-8 (IL-8), which is a cytokine (protein) produced by different cells in the body including immune cells and involved in the regulation of inflammation and the immune response.
- Tumour necrosis factor alpha (TNF-α), which is a cytokine (protein) produced by immune cells (macrophages, monocytes, etc.) and involved in the regulation of inflammation and the immune response.

In some of the in vivo studies assessed, the subjects’ behavioural changes were also noted, for example, the swimming frequency and pattern of fish. This information can be extremely insightful, by showing us how the compounds can affect the subjects’ nervous system, motor skills, and overall wellbeing. However, behavioural changes alone cannot give the full picture, and they should always be used in combination with toxicological tests. This is the case because behaviour can easily be affected by the stress and trauma caused to the subjects during the administration process; hence, there should always be precautions in place in order to minimise the stress caused. Some of these precautions include habituation, maintaining a constant environment, and proper handling. Nevertheless, in some cases, such as intratracheal installations, there is little that can be done in order to minimise the stress caused to the subject.

### 2.3. Methods for Brake Sample Collection

The main methods used to collect brake samples were as follows:

- Brake bench dynamometer.
- Box around brakes.
- Brake drum or brake lining of vehicles.
- Room filter of brake bench dynamometer installation.
- Grinding of brake pads.

It is crucial to take note of how the emission samples were collected in these studies in order to figure out if the results are realistic and representative of real life. Many of the brake wear particle samples were not collected using a representative methodology. In laboratory sampling, the conditions used were often extreme in order to collect the samples quickly; however, this means that the morphology and composition of the samples were not representative of real emissions. When the same brake pad goes through multiple braking cycles without appropriate pauses and ventilation, and the temperature and pressure are extremely high, physical and chemical reactions which normally do not take place can happen and possibly create new compounds. There are situations where these extreme conditions might occur; however, it is important to be realistic about the probability of the general population to be exposed to these types of particles, in order for them to cause harm. Most of these extreme driving conditions happen outside of urban environments, often in scarcely populated areas or on racing tracks. For example, wear cycles like AO4D and SAE J2522, which were designed to assess the effectiveness behaviour of a friction material, result in extreme driving conditions and high temperatures [46,47], which are unrealistic for passenger vehicles.

One of the most realistic sample collection methods, out of the ones mentioned in this review, was carried out in a bench dynamometer test chamber [48]. In this study, three different brake pads were tested (low-steel, high-steel, and NAO brake pads). All the brake pads were run through three different brake cycles: two realistic, or mild, conditions and one severe. The first cycle consisted of 8 braking events of mild deceleration, 120 to 80 km/h, and a temperature of 100 °C. The second cycle consisted of 8 braking events of more intense deceleration, 200 to 170 km/h, and a temperature of 100 °C. The last cycle consisted of 15 braking events of extreme deceleration, 100 to 5 km/h, and reached temperatures of 550 °C. The severe cycle reached extreme temperatures which would not normally occur during real-life driving conditions. However, it must be noted that the particles collected from all three cycles were then combined and the organic parts extracted. This means that, although two of the cycles run were realistic, the final sample contained a large proportion of particles from the extreme cycle and hence is not representative of the majority of real-life emissions. Furthermore, only the organic extracts were used for the toxicology assessment, so the results of this study cannot be considered representative of brake wear emissions as a whole.

In one 2009 paper [49], the particles used were freshly collected through an exposure box installed around one brake of a car. The cell lines used to assess the health effects of brake wear particles were placed directly inside the exposure box in order to simulate real-life exposure. This is a much more realistic way to assess the toxicity of fresh particles, which are usually the type of particle humans are exposed to in urban environments. However, in this specific study, the vehicle was installed on a garage lift, so the tyres were not touching the pavement, and the forces involved in the braking procedure were not necessarily realistic.

Another method used is collecting the sample from the brake drums of vehicles after prolonged use. For example, in one study, samples from the brake drums of seven passenger vehicles with an average age of 10 years and average distance of 51,732 km were collected [50]. Although this method is fairly realistic, it has two main flaws. Firstly, the samples collected are not necessarily representative of real emissions, as the brake drums are not completely closed and can allow for some of the smaller size fractions to escape, as well as let particles from other sources, like tyre and road wear or exhaust emissions, enter. Secondly, some of the particles collected were released a long time before sample collection, which means that these aged particles could potentially have a very different composition and morphology to the original emissions, due to processes like oxidation and agglomeration, skewing the results once again. Thus, the results of such studies on aged particles (e.g., [50]) may be different compared to other studies on fresh particles, probably due to the highly oxidised and aged state of the particles and their larger size. Nevertheless, research comparing aged and fresh brake particles is of interest. The researchers also collected samples from the brake linings of three different vehicles after use; however, not much more information is given about the sample collection.

Another study collected samples from the filter of a brake testing laboratory, containing a year’s worth of particles [51]. Like before, the samples used in this study might not be representative of real-life emissions, as the particles were aged and also because they came from a laboratory, through a variety of testing cycles and possibly unrealistic driving conditions.

Probably the least representative sample collection method is the grinding of brake materials. The particles released during real braking scenarios are different from the particles created by grinding the same materials. During typical braking behaviours, the temperatures rise significantly, and chemical reactions can occur, creating different compounds. This is shown in a series of studies [46,52] in which particles collected from a road simulator and ball-milled particles were both assessed. In both studies, the ball-milled samples caused a mutagenic response, whereas the friction samples did not cause any significant response, which further highlights the difference in the particles produced by different methodologies.

Out of the papers mentioned in this review, only four [52,53,54,55] carried out in vivo experiments, unfortunately using unrealistic sample collection, highlighting the need for more research on the topic. To this date, there has not been an in vivo study using a representative sample collection methodology published yet, in order for the results to be able to be taken into consideration when assessing the potential toxicity of brake wear particles. This means that although the results produced from these papers are important to take into account, they cannot be used to determine the real adverse effects on human health.

### 2.4. Methods for Tyre Sample Collection

The main methods used to collect tyre samples were as follows:

- Laboratory road simulator.
- Cryo-milling or cryo-fracturing of tyres.
- Grinding, shredding, or scraping of tyres.
- Pre-made crumb rubber (with unknown grinding technique).

The main challenge when it comes to collecting tyre emissions is that, unlike drum brakes, for example, the particles are not collected anywhere, and they are released directly into the environment, which makes it especially hard to collect them during on-road testing, which would be the most realistic. Laboratory testing with a road simulator consists of tyres mounted on a rotating axis and a round pavement for the tyres to run on. Although this method is convenient, it is not completely accurate, as the tyres are constantly at a slight angle, and there are no perpendicular forces applied like there are in real-life driving. This does not represent real-life driving conditions where the tyres are constantly changing direction. The representativeness of the particles emitted from tyres in a road simulator is also dependent on the conditions used during the testing and the pavement composition, although, unlike brakes, the temperatures reached are not extremely high, and so there should not be a significant difference.

Many studies rely on other methods to collect particles that are much faster and cheaper but, unfortunately, much less realistic. One of the methods used the most is cryo-milling or cryo-fracturing. This method consists of taking scraps or small parts of tyres and freezing them with liquid nitrogen in order to make them brittle, without altering their composition, and then ball milling them until they reach the desired particle size. Other similar methods are grinding, shredding, or scraping the tyre to create particles. Although these processes are very convenient, as they allow for a lot of control, they are not at all representative of the real-life process of tyre abrasion and the particles emitted into the environment. One more method used in some of the papers is rotating a tyre against a steel brush [56]. This method is also not completely accurate; however, it is slightly more realistic than grinding and ball milling, as the particles released are created by abrasion.

In all but three of the studies carried out on aquatic organisms, the samples used were not representative of real emissions, as most of the samples were obtained from cryo-milling or cryo-fracturing, shredding and grinding, or crumb rubber materials. And out of the studies carried out on terrestrial organisms, none of them were carried out using realistic sample collection methods. On the other hand, in the studies conducted in mammalians, eight of them used samples collected in a road simulator. In the studies carried out on aquatic organisms, the researchers seemed to value more the time and cost efficiency that techniques like cryo-milling provided, whereas in the studies carried out on mammalian organisms, the researchers seemed to want to obtain the most realistic results, in order to have an accurate representation of the health effects on humans.

In the aquatic studies, the only ones that used realistic sample collection were conducted by collecting samples from a road simulator laboratory in an interior drum testing system with asphalt pavement in cassettes [57]. Considering the fact that their studies are the only ones carried out with representative samples, it is interesting to see that the results only showed mild toxicity and no significant effects on the growth or reproduction of non-salmonid species.

In many of the studies conducted on aquatic organisms, the exposure was conducted through leachates, sediments, and elutriates. This is mostly performed because the exposure mechanism for aquatic organisms is usually through particles entering the water system. Many different leaching methodologies were used. One study even used sequential leaching to see how that would change the toxicity.

## 3. Results

**Figure 2:**
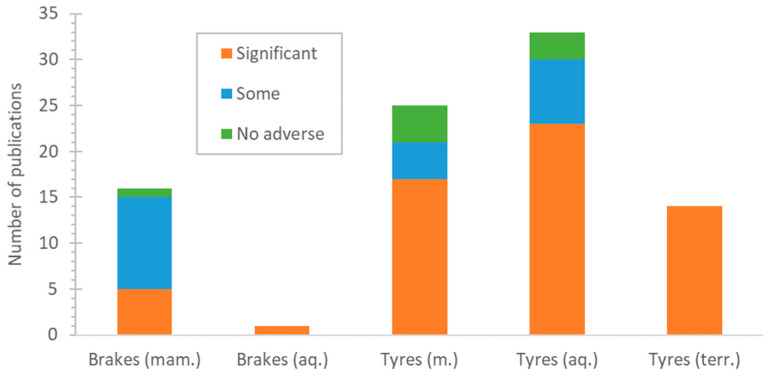
Number of publications that examined the impact of tyre particles on various cells. mam. = mammalian; aq. = aquatic; terr. = terrestrial.

Figure 2 summarises the studies and their conclusions regarding toxicity. The papers were separated into the ones that showed no adverse health effects, the ones that presented few health effects or not extremely harmful ones, and the ones that showed significant or detrimental adverse effects on the organisms studied.

### 3.1. Brakes

Table A1 of Appendix A summarises the studies that examined brake particles. More details about the studies can also be found in Appendix A. From the sixteen mammalian studies assessed, one showed no effects [46] (the ball-milled samples were mutagenic but not the friction samples), five showed significant effects, and ten showed some effect. Overall, the three studies that used realistic testing methodologies [49,50,58], i.e., sample collection from drum brakes after prolonged use, all showed some toxicological effects and pro-inflammatory responses, mostly ROS production and IL-8 secretion. It is important to note that most of the studies observed a concentration-dependent or dose-dependent response. For example, a study that exposed cells to fresh particles through an exposure box noted an increase in IL-8 levels with an increasing number of braking events and strength [49].

Another study suggested that the overall oxidative potential of brake wear particles can lower over time, since the ROS generation they observed studying aged brake wear particles (average 10 years) was lower compared to their previous study that tested fresh particles [50]. This is probably due to the highly oxidised state of these aged particles and because of their larger size, due to agglomeration, and smaller reactive surface area.

Many studies seem to suggest that the toxicity of brake wear particles is partly due to the presence of certain metals and heavy metals, known to be cytotoxic. This is further validated by the use of metal chelators, which bind to the metal ions forming a complex and removing the metals from the system. A study observed that after PM exposure, in the presence of a metal chelator, the initial heightened IL-8 and TNF-α secretion, as well as the impairment of phagocytic capacity, was completely abolished [51]. Furthermore, in a 2020 study, brake pads with different Cu concentrations were tested [59]. A correlation between Cu concentration and cell toxicity was found. However, it should be noted that it is extremely hard to prove that the toxicity is caused specifically by one component of the particles. For example, in one study, it was not possible to correlate the toxicological effects with the composition of the particles of low-steel, high-steel, and NAO brake pad samples [48].

When looking at the studies that did not use representative sample collection methods (e.g., [54]), most of them produced mixed results with no clear verdict on whether or not the particles produce significant toxicological responses. Overall, it is evident that brake wear particles are not intrinsically toxic; however, the size, composition, and exposure dose are what makes them dangerous to human health.

Some studies tested only asbestos-based brakes, which have been banned in Europe and the United States of America (USA) since the late 1990s due to health concerns [53]. Although not relevant to EU policy, there are, however, many countries that still use asbestos-based brake pads and linings to this day. Studies on asbestos-based brake materials confirmed what was already known about asbestos materials. Brake dust containing chrysotile showed no to little pathological response, as it is biosoluble and was able to be cleared from the lungs. On the other hand, crocidolite- and amosite-containing brake dust caused significant inflammation and a pathological response, as the fibres were not able to be cleared from the lungs. This happens because chrysotile particles are long and curly, whereas crocidolite and amosite fibres are known for their short and pointy shape, which makes them much more of a hazard, showing that the shape and size of a particle can make it more dangerous to human health.

### 3.2. Tyres

Table A2 summarises the studies that examined tyre particles in human/mammalian, aquatic, and terrestrial cells. More details about the studies can be found in Appendix A.

#### 3.2.1. Human/Mammalian

In the studies conducted on mammalians, there was a big variety of sample collection and testing methodologies; hence, it can be difficult to compare results. Overall, three of the studies showed no significant effect caused by tyre wear particles [57,60,61]. A fourth in vitro study did not follow an up-to-standard methodology: only two strains of bacteria were used, even though, according to the OECD (Organisation for Economic Co-operation and Development) guidelines, at least five strains should be used [62]. Four studies showed mild effects [45,63,64,65], while the remaining seventeen studies showed significant toxicity. Out of the four studies that showed no effects, three were carried out in vitro. Additionally, out of these four studies, three of them were conducted using samples collected from a road simulator. However, as there were both in vitro studies and studies with road simulator samples that showed effects, no conclusion can be drawn regarding the non-reliability of any of these studies.

Some of the studies that used samples from a road simulator used different types of pavements during the sample creation, which affected the particles created, as a big proportion of the particles originated from the pavement surface. Overall, both granite and quartzite pavements were able to cause a toxicological response; however, granite caused a much stronger response. Granite caused higher cytokine release, and although both pavements induced similar lipid peroxidation and ROS formation, only granite induced nitrogen oxide (NO) production [66,67,68]. However, it should be noted that these studies used studded tyres, which are representative only of winter conditions in Scandinavian countries.

One study [69] used a three-dimensional (3D) human airway organoid made from human bronchial epithelial cells in order to assess the toxicity of tyre wear particles. This in vitro model can give a much more accurate overview of the real response that might occur in human airways when exposed to harmful particles. In this study, the samples were able to induce significant cytotoxicity at concentrations higher than 200 μg/mL, as well as significant ROS generation in a dose-dependent manner. An increase in oxidative stress-related and cytokine-related gene expressions including TNF-α, IL-6, and ccl2 was observed. Interestingly, the larger particles were found to be clustered around the epithelial cells, and the smaller particles were found inside the cells. This type of information was only observed because of the 3D model.

After the discovery that the by-products of tyre wear 6PPD (N-(1,3-dimethylbutyl)-N′-phenyl-p-phenylenediamine) and 6PPDQ (N-(1,3-dimethylbutyl)-N′-phenyl-p-phenylenediamine quinone) are toxic to coho salmon in 2020, much more research has been conducted in order to assess their toxicity to marine organisms, with over 25 papers about the effects of tyre wear particles on aquatic life being published since 2021. This will be discussed in the next section. In comparison, only 10 new papers about the health effects of tyres on mammalians have been published since 2021, to our knowledge. There are only two mammalian papers that studied the effects of 6PPD and 6PPDQ in mice [70,71]. Both papers found the substances to be bioaccumulative in the liver of mice in a dose-dependent manner. Exposure doses and frequencies seem to have a strong effect on the toxicity of these compounds [71], where a single injection did not cause any significant changes in the mice; however, repeated exposures were able to cause significant changes in biochemical parameters in multiple organs.

One more paper mentioned the existence of 6PPD and 6PPDQ in mammalians [72]. In this 2022 paper, the researchers conducted a biomonitoring study on humans, where they monitored the urine concentrations of these substances in three human populations: adults, children, and pregnant women. Both 6PPD and 6PPDQ were detected at frequencies between 60 and 100%, although the 6PPDQ concentrations were significantly higher. Interestingly, both concentrations were much higher in pregnant women compared to adults and children. After in vitro metabolic experiments, the results showed a rapid depletion of 6PPD by human liver microsomes, which could be responsible for the lower concentration of 6PPD. Considering all the research showing the extensive toxicological effects of these substances in other organisms, the fact that there are significant concentrations found in human urine should be a cause for worry, and it should push for more research to be conducted in this field.

#### 3.2.2. Aquatic

Out of the thirty-three studies conducted on aquatic organisms, only three of them found no significant effects caused by tyre wear particles [73,74,75], while seven of them found some effect, and the remaining twenty-three showed significant effects. However, as these studies were all conducted in different organisms, it is hard to establish the particles’ toxicity.

As previously mentioned, a few years ago, there was a sudden interest in the tyre by-products 6PPD and 6PPDQ, after the discovery of their effects on coho salmon. Although at the beginning this finding was seen as a threat to all salmonid species, after more research, it was established that the toxicity primarily affected coho salmon. In fact, in a 2021 paper [76], the effects of tyre particles on coho and chum salmon were assessed, and the results showed that while coho salmon displayed significant behavioural changes, changes in blood parameters, and mortality, chum salmon seemed to be unaffected by exposure. This further confirms that the toxicity of tyre wear particles varies significantly between animals and even between species.

In another paper [77], the toxicity of tyre particles was assessed using samples that had been affected by different stressors. In this study, the researchers used two tyre samples that had been exposed to different levels of four physical stressors (temperature, mechanical stress, ultraviolet (UV) light, and CO_2_). In the temperature experiments, there was no significant effect on time to hatch; however, hatching success decreased in the second-tyre experiments. Significant differences in heart rates were observed for different materials and different temperatures. Increasing deformities and shorter lengths were observed with increasing temperature, although the second tyre caused more severe deformities. Both mechanical stress and UV exposure caused very similar effects to temperature changes, where the effects were consistent between the two tyre types, although once again, the second tyre caused more severe effects. The effects of carbon dioxide were not significant enough to influence the leaching process. Overall, the results showed that the variations in temperature and mechanical stress caused a significant change in toxicity, whereas UV and CO_2_ exposure caused milder effects. This study was able to show how the toxicity of particles can easily change through different stressors and, hence, why particles in real life, which are exposed to such stressors, can potentially be much more harmful than laboratory-made tyre wear particles.

Another paper in 2024 looked at different tyre particles after being put through stressors [78]. In this paper, the particles were exposed to UV irradiation and two different doses of K_2_S_2_O_8_ in order to simulate short- and long-term aging. The results showed that aged particles exhibited enhanced toxicity towards microalgae, compared to virgin particles, because of their increased leaching potential and physiochemical damage. Moreover, the exposure to particles and their leachates altered the metabolic profiles, and especially UV- and high-dose-K_2_S_2_O_8_-aged particles caused the biggest effect.

One study in 2009 assessed the toxicity of sequential tyre wear particle leachates [79]. Particles were placed in purified water multiple times in order to retrieve sequential leachates. The results showed that the sequential leachates were much less toxic than the original samples. This effect is most likely caused by the dilution that occurs after multiple leachings, making the final exposure concentrations much smaller and, hence, much less toxic.

#### 3.2.3. Terrestrial Invertebrates

We found fourteen studies that were carried out on terrestrial organisms (one more on a bacterial community), mostly invertebrates and crustaceans. All of these studies used samples created in laboratories by cryo-milling or grating or crumb rubber. They all showed significant effects on the organisms studied; however, this could be because of higher exposures causing more toxicity. Overall, there are too few studies on terrestrial organisms, and more research needs to be carried out.

### 3.3. Species

**Figure 3:**
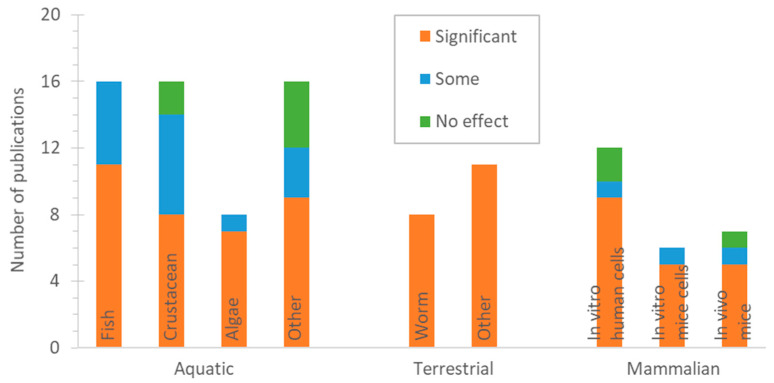
Number of publications that examined impact of tyre particles based on species.

Figure 3 summarises the number of studies that assessed the impact of tyre particles on different species. There is no clear tendency of a particular species being affected less or more by tyre particles.

### 3.4. Non-Exhaust vs. Other Particles

**Figure 4:**
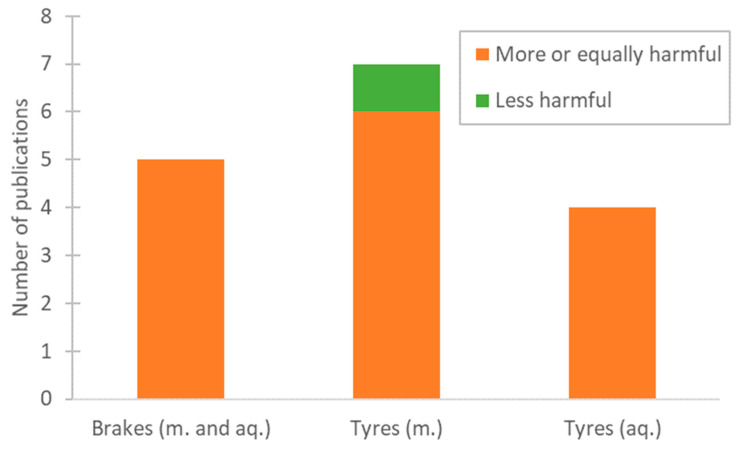
Number of publications that compared non-exhaust particles with other sources of particles.

From all the papers analysed, sixteen of them compared non-exhaust emissions to other sources of particulate matter (Figure 4). Although it is hard to draw a direct comparison, as it is important to take the whole exposure pathway into consideration, it is, nevertheless, interesting to analyse the different sources of PM in order to have a more comprehensive view of the problem at hand.

Out of the papers on brake emissions, five of them discussed other sources of pollution [48,51,58,80,81]. Generally, it seems that brake particles are at least as toxic as the other sources tested, like urban dust and diesel and wood combustion. In a 2023 study, it was revealed that, although all the sources studied were found to be genotoxic, brake dust was the most cytotoxic [58]. Another study stated that brake pads caused the greatest decrease in cell viability among the sources tested [48].

From the papers on tyre emissions conducted on mammalians, seven of them [45,60,61,66,67,68,82] assessed other sources and compared them to tyre particles; interestingly, most of them were carried out using samples from studded tyres [60,66,67,68,82]. All concluded that the airway inflammatory and genotoxic potential of particles from studded tyres is at least equal, if not stronger, than that of other sources, more specifically, diesel particles. Most of the papers also agreed that subway particles caused a stronger response than any of the other sources tested. Lastly, one paper [61] showed no effect on the 3D intestine barrier model used in the study, for both tyre particles and the other microplastics tested.

On the other hand, out of the papers on the effects of tyre emissions on aquatic organisms, there were only four [83,84,85,86] that compared them with other sources, namely other microplastics and other non-traffic-related emissions. All four papers agreed that the tyre emissions had a more significant effect on the aquatic organisms tested, compared to polyethylene terephthalate (PET), polystyrene, polyester, polypropylene, and polyvinyl chloride (PVC).

Overall, most of the papers agreed that non-exhaust emissions are comparable to other sources like exhaust emissions and wood combustion. Some of the other sources assessed in these papers were poultry farms, polyester fibres, polystyrene microplastics, carbon nanotubes, diesel exhaust particles, coke dust, and pellet ashes. These findings prove that there should be a much more serious effort towards the regulation of non-exhaust emissions, considering that many of the other sources of pollution discussed, which are currently regulated in order to improve human health, are comparable to emissions from tyres and brakes, in term of toxicity.

### 3.5. Samples

It is crucial to highlight the lack of research on certain specific types of tyres and brakes. Table A3 and Table A4 in Appendix A show a clear lack of toxicological studies carried out on certain types of non-exhaust emissions. One of the most evident gaps in this review is the lack of research on heavy-duty vehicles, with only five studies using heavy-duty brakes or tyres in their toxicological assessment. Another noteworthy issue is the amount of research conducted on additives and extracts from tyres, as well as non-specified tyre and rubber materials, instead of real tyres.

There is a clear difference in composition between summer and winter tyres and, hence, in the composition of the particles released. This difference is even more significant for studded tyres which use tungsten carbide studs. This is why it is important to take into account the types of tyres used while assessing their abrasion. Moreover, heavy-duty tyres also have considerable differences in their composition. Similarly, brake systems vary immensely in their composition and functionality. There are non-negligible differences between markets and between uses.

## 4. Conclusions

This review paper gave an overview of the literature on the health effects of non-exhaust emissions and highlighted the gaps in the current research. Overall, most of the papers agree that particles from non-exhaust sources can cause adverse health effects. Almost all of the studies saw some toxicity; however, many of them showed that the effects can be extremely detrimental and should be a cause to worry. Some of the main issues seen were changes in inflammation and organ parameters like ROS, IL-8, and TNF-α, as well as issues in the lung and airway tissues. In some cases, the impact was attributed to heavy metals like zinc and copper, PAHs, 6PPDQ, dithiobisbenzanilide (DTBBA), 2-mercaptobenzothiazole (MBT), and other chemicals, however, without being able to establish a causal relationship. Studies that compared non-exhaust particles with other particles (subway, urban environment, and exhaust particles for mammalians or PET/PS for aquatic cells) found that tyres and brakes are in general more harmful.

Even though there are many toxicological studies in the literature, there is a need for further research in order to have a full picture of the real effects of tyre and brake emissions. One of the main concerns with the current literature is that in many cases the samples used in the toxicological assessment were created in the laboratory in a way that is not representative of real-life emissions. This means that the results of these studies are not necessarily representative of the real health effects caused to humans when exposed to such particles. It is important to understand at what levels non-exhaust particles become (or stop being) harmful and compare those with measured concentrations in various environmental compartments. One important thing to note is the lack of toxicological research for certain types of non-exhaust emissions, like those derived from heavy-duty vehicles. Another significant issue in this area is the lack of epidemiological studies, which are the golden standard when it comes to health research.

## Figures and Tables

**Table 1:** Reviews on health impacts of non-exhaust particles.

Year	Ref.	Brakes	Tyres	Comments
2018	[9]	N	Y	Aquatic environment
2020	[31]	N	Y	Human effects
2022	[32]	Y	N	Human effects
2023	[33]	Y	N	On mammalian models
2023	[24]	N	Y	In vivo and in vitro studies
2023	[34]	N	Y	Only urban parks
2024	[35]	N	Y	Environmental impact
2024	[22]	N	Y	Mostly ecological and ecotoxicological
2024	[36]	N	Y	Leachates
2024	[37]	N	Y	Aquatic and terrestrial effects
2024	[38]	N	Y	Environmental and health

## Data Availability

Data are contained within this article.

## Abbreviations

3D	Three-dimensional
6PPD	N-(1,3-dimethylbutyl)-N′-phenyl-1,4-phenylenediamine
6PPDQ	6PPD quinone
ABC	ATP binding cassette
ABS	Stone mastic asphalt
ABT	Asphalt concrete
AChE	Acetyl-cholinesterase
ALP	Alkaline phosphatase
ALT	Alanine aminotransferase
ARG	Antibiotic-resistant gene
AST	Aspartate aminotransferase
ATP	Adenosine triphosphate
BALF	Bronchoalveolar lavage fluid
BPA	Bisphenol A
BPAF	Bisphenol AF
BPF	Bisphenol F
BPS	Bisphenol S
BT	Benzothiazole
Car	Carotenoids
CAT	Catalase
CAV-1	Caveolin-1
CD55	Lipid raft marker
Chla	Chlorophyll a
DCFH-DA	2′7′-dichlorodihydrofluorescein diacetate
DEGs	Differentially expressed genes
DFO	Deferoxamine
DFX	Deferoxamine mesylate
DNA	Deoxyribonucleic acid
DPG	1,3-diphenylguanidine
DRM	Detergent-resistant membrane
DTBBA	Dithiobisbenzanilide
EC50	50% effect concentration
ECE	Economic Commission for Europe
EDTA	Ethylene diammine tetra acetic acid
EPFRs	Environmentally persistent free radicals
EU	European Union
FETAX	Frog embryo teratogenesis assay—Xenopus
FLOT-1	Flottilin-1
FTIR	Fourier-transform infrared spectroscopy
GGT	γ -glutamyl transpeptidase
GO	Gene ontology
GPx	Glutathione peroxidase
GSH	Glutathione
GST	Glutathione S-transferase
hAO	Human airway organoid
HO-1	Heme oxygenase-1
Hsp70	Heat shock protein 70
IL-8	Interleukine-8
LC50	Lethal concentration for 50% of the test subjects
LDH	Lactate dehydrogenase
LM	Low-metallic
LMS	Lysosomal membrane stability
LOEC	Lowest observed effect concentration
MBT	2-mercaptobenzothiazole
MDA	Malondialdehyde
MDDC	Monocyte-derived dendritic cell
MDM	Monocyte-derived macrophage
MEF	Membrane-enriched fraction
MIP-2	Macrophage inflammatory protein-2
MMI	Methimazole
mRNA	Messenger ribonucleic acid
MTT	3-(4,5-dimethylthiazol-2-yl)-2,5-diphenyltetrazolium bromide
NAG	β-N-acetylglucosaminidase
NAO	Non-asbestos organic
NO	Nitrogen oxide
NOEC	Non-observable effect concentration
OECD	Organisation for Economic Co-operation and Development
PAHs	Polycyclic aromatic hydrocarbons
PEF	Peak expiratory flow
PET	Polyethylene terephthalate
PM	Particulate matter
PMN	Polymorphonuclear neutrophilic leukocyte
PVC	Polyvinyl chloride
ROS	Reactive oxygen species
SOD	Superoxide dismutase
SM	Semi-metallic
TBARSs	Thiobarbituric acid reactive substances
TC50	Teratogenic concentration for 50% of a test species
TIE	Toxicity identification evaluation
TNF-α	Tumour necrosis factor alpha
TOF-MS	Time-of-flight mass spectrometry
TPs	Tyre particles
TPO	Thyroid peroxidase
TRWPs	Tyre and road wear particles
TWPs	Tyre wear particles
USA	United States of America
UV	Ultraviolet
VACES	Versatile ambient concentration enrichment system
VTI	Swedish National Road and Transport Research Institute
WHO	World Health Organization

## Appendix A

### Appendix A.1. Brakes

The following section presents the studies that investigated the impact of brake particles on health. For each study, a short summary is given. All studies were carried out on human/mammalian cells, except one on aquatic organisms, which is given at the end of this section.

#### Appendix A.1.1. Human/Mammalian

**2009: Wear Mechanism in Automotive Brake Materials, Wear Debris and its Potential Environmental Impact** [52].

This study assessed the mutagenicity and pulmonary toxicity of brake wear debris in vitro through two bacterial microbioassays and in vivo through a pulmonary toxicity test on rats. The samples were collected from a model semi-metallic (SM) brake pad run through a modified A04D dynamometer test and from ball-milled brake wear debris, which are not representative of real driving conditions. The experiments showed a possible mutagenicity for the milled brake pads with metabolic activation and a clearance response of alveolar macrophages depending on the exposure dose.

**2009: Toxic Effects of Brake Wear Particles on Epithelial Lung Cells In-Vitro** [49].

In this study, the toxicological effects of brake wear particles on epithelial lung cells exposed to freshly generated particles were evaluated in vitro. The cell lines were exposed to the particles through an exposure box fitted around a car’s braking system, while two different typical braking behaviours (full stop and normal deceleration) were performed. The study showed that the particles did not cause any cytotoxic response; however, some oxidative stress was observed with full-stop braking with multiple repetitions. IL-8 levels also increased with an increasing number of braking events and strength, which shows the presence of a pro-inflammatory response.

**2015: Physico-Chemical Characterization and Oxidative Reactivity Evaluation of Aged Brake Wear Particles** [50].

In this study, the ability of aged brake wear particles to generate ROS was assessed acellularly and cellularly in vitro. The samples were collected, without scraping, from the brake drums of seven small passenger vehicles with an average age of 10 years and average driving distance of 51,732 km. The acellular ROS generation of these particles was higher than that tested cellularly; however, the overall oxidative potential of aged brake wear particles was found to be lower than that of fresh particles tested in other studies. This is probably due to the highly oxidised and aged state of the particles and their larger size and smaller reactive surface area.

**2015: Evaluation of the Fate and Pathological Response in the Lung and Pleura of Brake Dust Alone and in Combination with Added Chrysotile Compared to Crocidolite Asbestos Following Short-Term Inhalation Exposure** [53].

This study assessed the pathological response to asbestos-based brake dust exposure in rats in vivo. One plain brake dust sample, one brake dust mixed with chrysotile sample, and one crocidolite asbestos sample were investigated during this study. The brake dust samples were collected by grinding chrysotile-containing brake drums. The crocidolite asbestos fibres were persistent through the lifetime of the animal and produced an inflammatory response in the lung, whereas the brake dust alone and brake dust with chrysotile samples were biosoluble and caused no significant pathological response.

**2016: Automotive Airborne Brake Wear Debris Nanoparticles and Cytokinesis-Block Micronucleus Assay in Peripheral Blood Lymphocytes: A Pilot Study** [47].

In this study, the genotoxicity of brake wear particles was assessed through the in vitro testing of human lymphocytes (blood cells). The airborne samples were collected in a laboratory from a brake dynamometer (cycle SAE J 2522, which is not representative of real driving conditions), which produced ultrafine PM. The testing showed an increase in the frequency of micronucleated binucleated cells in those exposed to brake wear particles for 48 h, which is likely due to the crystalline metal nanoparticles included in the sample.

**2016: Toxicity and Mutagenicity of Low-Metallic (LM) Automotive Brake Pad Materials** [46].

In this paper, the toxicity and mutagenicity of LM brakes was studied in vitro through one bioluminescence assay and two bacterial bioassays. Four types of samples were used during the experiment: two commercial LM automotive brake pads, a model brake pad with a known composition resembling typical LM brake pads (with extra phenolic resin in order to address the organic constituents of brake pads), non-airborne wear debris generated by a simulation of different braking scenarios using the model material, and phenolic acid and antimony trisulfide representing two potentially harmful components of brake pads. The two commercial brake pads and the model brake pad were also ball-milled and analysed. The ball-milled samples are unrelated to the friction process occurring during braking. The ball-milled samples were found to be mutagenic; however, none of the friction samples had a mutagenic response. The phenolic resin and wear debris samples were found to be acutely toxic.

**2018: Evaluation of the Dose-Response and Fate in the Lung and Pleura of Chrysotile-Containing Brake Dust Compared to Chrysotile or Crocidolite Asbestos in a 28-Day Quantitative Inhalation Toxicology Study** [54].

During this study, a series of in vivo experiments were conducted on rats after 28-day inhalation in order to assess the health effects of asbestos-based (chrysotile-containing brake dust, chrysotile, crocidolite asbestos) friction materials on human lungs and to prepare for a subsequent 90-day study. The brake dust samples were collected by grinding brake shoes, which is not representative of real-life driving emissions, whereas the chrysotile and crocidolite asbestos fibre samples were ordered directly from a supplier. The chrysotile samples caused a slight inflammatory response; however, as the fibres are biodegradable, they were able to be cleared by alveolar macrophage clearance. On the other hand, the crocidolite fibres were not able to be cleared, and they caused a significant inflammatory response and severe mesothelial pathology. The brake dust samples showed no significant pathological response.

**2018: Biological Response of an In-Vitro Human 3D Lung Cell Model Exposed to Brake Wear Debris Varies Based on Brake Pad Formulation** [87].

In this paper, LM and non-asbestos organic (NAO) brake pads were used to collect non-airborne PM_2.5_ samples, which were then used to study their effects on human lung cells in vitro. The samples were collected in a laboratory by simulating urban driving conditions (˂80 km/h, 200–500 °C) in a brake dynamometer. A 3D multicellular model of the human alveolar epithelial tissue barrier was used to assess the viability, morphology, oxidative stress, and pro-inflammatory response of the cells when exposed to the PM_2.5_ samples. Although the LM particles did not cause a biological response, the non-asbestos organic particles caused an inflammatory response, reduced the cell viability, and altered the cell morphology, likely due to the anatase present in the samples.

**2018: Brake Wear (Nano)Particle Characterization and Toxicity on Airway Epithelial Cells In-Vitro** [88].

In this study, brake wear particles were used to assess their toxicity in human lung epithelial cells in vitro. Various test cycles were carried out at a motor vehicle testing centre for light-duty vehicles and a test bench working with an automotive brake dynamometer. The brake systems were opened to collect the samples using a clean and sterile spatula, without scraping the disc and pad surfaces. The results showed a clear short-term loss of viability associated with ROS generation but with limited pro-inflammatory effects. The cytotoxicity of the nanosized fraction was found to be similar to that of brake wear particles, suggesting the cytotoxicity is particle-size-dependent.

**2019: Inhalation Toxicity Profiles of Particulate Matter: A Comparison Between Brake Wear With Other Sources of Emission** [80].

In this in vivo study, the effects of PM_2.5_ particles derived from different sources were analysed. The samples were collected in-lab using the versatile ambient concentration enrichment system (VACES) from different types of brake pads (“realistic driving” simulator at Technical University of Ilmenau), studded winter tyres (real-life representative at the Swedish National Road and Transport Research Institute (VTI) road simulator), Euro 3 diesel engine exhaust, and others. Mice were exposed to the aerosolised particle suspensions at different doses. No cytotoxicity or oxidative stress was observed; however, the potency required to induce inflammatory responses was different for the various sources. Through the analysis of lung inflammation, it was concluded that NAO and Economic Commission for Europe (ECE)-compliant and NAO hybrid brake pads possess a higher toxic potential compared to the studied LM and SM pads or to tyre/road wear particles; however, this could not be proven through the analysis of biomarkers for toxicity in the blood.

**2020: Brake Dust Exposure Exacerbates Inflammation and Transiently Compromises Phagocytosis in Macrophages** [51].

In this paper, samples from brake abrasion dust and diesel exhaust particles were tested through a series of in vitro experiments in order to assess their toxicity to human airways. The brake dust samples were collected from a measurement laboratory in Sweden and contained a year’s worth of abrasion dust from heavy-duty drum brakes in different driving conditions. Both particle sources caused similar toxicological effects, and for both particle sources, when in the presence of a metal chelator deferoxamine (DFO), the heightened IL-8 and TNF-α secretion and the impairment of phagocytic capacity were abolished, suggesting that the cause of these toxicological effects is dependent on the metal content of the particles.

**2020: Copper-Dependent Biological Effects of Particulate Matter Produced by Brake Systems on Lung Alveolar Cells** [59].

In this in vitro study, brake pads with different Cu concentrations were used to analyse the health effects and correlate them to the concentration of copper. The samples were collected from four different brake pad/disc combinations during two 15 min braking bench tests (no details are provided). The smallest Cu concentration studied did not cause significant toxicological effects, except from ROS production and IL6 gene expression; however, the higher Cu concentrations were found to cause cell toxicity dependent on the Cu concentration.

**2021: Final Results from a 90-day Quantitative Inhalation Toxicology Study Evaluating the Dose-Response and Fate in the Lung and Pleura of Chrysotile-Containing Brake Dust Compared to TiO_2_, Chrysotile, Crocidolite or Amosite Asbestos: Histopathological Examination, Confocal Microscopy and Collagen Quantification of the Lung and Pleural Cavity [55].**

In this paper, the toxicological effects of different asbestos-based brakes were assessed in a 90-day inhalation exposure toxicological in vivo study on mice, with lifetime post-exposure observations. During the study, different combinations of chrysotile-containing brake dust with chrysotile fibres, crocidolite asbestos, amosite asbestos, and titanium dioxide were used. The brake dust particles were produced by grinding brake shoes, which is not representative of actual braking. Brake dust and chrysotile showed no significant pathological or tumourigenic response; however, crocidolite and amosite showed inflammation, microgranulomas, persistent fibrosis, and a dose-related lung tumour response.

**2023: Effects of Particle-Bound Polycyclic Aromatic Hydrocarbons and Plasticisers from Different Traffic Sources on the Human Alveolar Epithelial Cell Line A549** [48].

During this study, the health effects of the organic extracts (PAHs and plasticisers) of brake wear, diesel exhaust, and road tunnel particles were studied in vitro through a series of experiments. The brake wear samples were collected in a bench dynamometer test chamber using low-steel, high-steel, and NAO brake pads, during two realistic braking cycles and one severe braking cycle. The brake wear samples did show some cytotoxicity and decreased cell viability; however, no linear relationship was established between the samples tested, which could indicate the effects to be caused by other constituents, like metals. The brake wear samples caused no increase in intracellular ROS and did not induce cell cycle arrest.

**2023: Effects of Brake Wear Nanoparticles on the Protection and Repair Functions of The Airway Epithelium** [89].

Multiple in vitro experiments were run in this study in order to assess the toxicity of brake wear nanoparticles. Samples were collected from a motor vehicle testing centre. The tests showed that, even through acute exposure, there was translocation through the epithelial barrier and increased mucus production but no cytotoxicity or inflammatory response.

**2023: Toxicological Profile of PM from Different Sources in the Bronchial Epithelial Cell Line BEAS-2B** [58].

In this paper, the cytotoxicity, genotoxicity, and oxidative and inflammatory responses were assessed in vitro in a bronchial cell line, for a few different PM sources, including diesel exhaust particles and brake dust. The brake dust samples were collected from the brake linings of three different vehicles. The study concluded that, although all the tested samples showed genotoxic action, the brake dust had the biggest cytotoxicity. Brake dust showed a reduced cell viability in all the concentrations tested and significant secretion of the pro-inflammatory cytokine IL-8. The authors hypothesise that the toxic character of the brake dust PMs is linked to their rich concentration of metals, such as Fe and Cu, and heavy metals, such as Mn and Cd, which are known to be cytotoxic.

#### Appendix A.1.2. Aquatic

**2017: In-vivo Assessment of the Genotoxic and Oxidative Stress Effects of Particulate Matter on *Echinogammarus veneris*** [81].

In this study, seven types of atmospheric dust, including brake dust, were used to assess their genotoxicity in *Echinogammarus veneris* in vivo. The brake dust samples were collected from the brake linings of three different vehicles. All the dust samples tested caused significant non-specific deoxyribonucleic acid (DNA) damage, and dust samples with a high concentration of elements, like brake dust, showed oxidative stress.

### Appendix A.2. Tyres

The following section presents the studies that investigated the impact of tyre particles on health. For each study, a short summary is given. The studies are sub-divided depending on the receptor into the following:

- Human/mammalian;
- Aquatic;
- Terrestrial.

#### Appendix A.2.1. Human/Mammalian

**2005: Impact of Tire Debris on In-Vitro and In-Vivo Systems** [90].

In this paper, the health effects of tyre debris eluates and organic extracts were studied in vitro and in vivo through a series of experiments. After analysing the particle morphology, tyre debris eluates and organic extracts were tested at different concentrations and exposure times on human cells and *Xenopus laevis* embryos. The samples were collected from a laboratory degradation test using the cryo-fracturing technique with tyre scraps.

Fourier-transform infrared spectroscopy (FTIR) analysis showed that the organic extract obtained from the Soxhlet apparatus is mostly composed of isoprene, which is known to be tumourigenic to mice. When assessing A549 cells, the tyre debris extracts were shown to cause an increase in DNA damage and a decrease in cell proliferation in a dose-dependent manner, especially for higher doses (50, 60, 75 μg/mL), as well as morphological changes more evident at 72 h of treatment, such as visible vacuolisation in the cytoplasm and apoptotic nuclear images. An increase in the mortality rate of *X. laevis* embryos was observed; however, the toxicity of 50 g/L was three times greater than that of 100 g/L, probably due to the aggregation of particles causing a lower surface area and hence less interactions between the particles and the medium. An increase in the percentage of malformed larvae was also observed but at similar percentages for both 50 g/L and 100 g/L. Lastly, a time-dependent increase in intracellular Zn was observed in HepG2 cells.

**2005: Toxicity of Tire Debris Extracts on Human Lung Cell Line A549** [56].

In this study, the effects of tyre debris on human lungs and its cytotoxicity were assessed through in vitro experiments on A549 human alveolar cells, using tyre debris organic extracts. The tyre samples were collected in a laboratory by rotating a new tyre against a steel brush, trying to mimic the normal wear of a tyre.

FTIR showed that the organic extract obtained from the Soxhlet apparatus is mostly composed of isoprene, which is known to be tumourigenic to mice. The results showed a significant dose- and time-dependent inhibitory effect on the reduction of (3-(4,5-dimethylthiazol-2-yl)-2,5-diphenyltetrazolium bromide) MTT (assay) at 48 and 72 h and a dose-dependent increase in cell mortality. A dose-dependent increase in DNA strand breakage and apoptotic or necrotic cells were observed at 24 and 72 h. The cell cycle analysis showed a significant increase in the percentage of cells in the G1 phase at 24 h of exposure, associated with a decrease in the percentage of cells in the S phase, whereas the increase at 48 and 72 h was associated with a decrease in the percentage of cells in both the S and G2/M phases. Multiple structural alterations were present in 50% of the cells at 75 μg/mL at 72 h.

**2006: Exposure to Wear Particles Generated from Studded Tires and Pavement Induces Inflammatory Cytokine Release from Human Macrophages** [66].

In this paper, the inflammatory effects of PM_10_ generated from studded tyres on two different types of pavements, compared to PM_10_ from a traffic-intensive street, subway station, and diesel exhaust particles, were investigated in vitro on human monocyte-derived macrophages, nasal epithelial cells, and bronchial epithelial cells. The tyre and pavement wear particle samples were collected from the VTI road simulator using studded tyres and two types of pavements, dense asphalt with granite and stone mastic asphalt with quartzite, which means that there cannot be a direct comparison between granite and quartzite. Cells were exposed to 10, 50, 100, 250, or 500 μg/mL of suspended particles.

Macrophages showed a significant pro-inflammatory cytokine release as a response to all types of particles used, and tyre particles released cytokines around the same magnitude or higher than the other sources tested, although the granite pavement caused a much higher release compared to the quartzite pavement. The authors believe this could be because of a variety of different reasons, including differences in the chemical composition, size and shape of particles, and resistance to fragmentation. Bronchial epithelial cells showed significant TNF-α secretion for all types of particles tested. Both granite and quartzite were able to decrease the cell viability at 500 μg/mL, whereas the street and subway particles showed significantly higher cytotoxicity. None of the particles were able to cause a cytokine release from the nasal epithelial cells.

**2006: Comparison of Genotoxic and Inflammatory Effects of Particles Generated by Wood Combustion, a Road Simulator and Collected from Street and Subway** [82].

In this in vitro study, the genotoxicity and inflammatory effects of studded and winter tyres on human alveolar cells A549 were assessed. The tyre wear samples were collected from the VTI road simulator at 30, 50, and 70 km/h, using one studded tyre on ABT (asphalt concrete) pavement, two studded tyres on stone mastic asphalt (ABS) pavement, and one friction (winter) tyre on sanded ABS pavement.

The results showed that all the particles generated in the road simulator were genotoxic. There was no significant difference between the samples collected from studded tyres on ABT or ABS pavement and between the different size fractions, i.e., PM_10_ or PM_2.5_; however, the samples collected from friction tyres on sanded ABS pavement caused much higher DNA damage compared to the other tyres.

Since only the studded tyre on ABT pavement was collected using a glass fibre filter, this was the only tyre to be assessed when testing the induction of cytokines of particles collected in glass fibre filters. This sample was able to increase the levels of IL-8 and TNF-α; however, the increase was much less significant than the urban street sample. The other tyre samples were collected on a Teflon filter. The studded tyres on ABS pavement caused a significant increase in all three cytokines tested (IL-6, IL-8, TNF-α), while the friction tyres on sanded ABS pavement did not induce an increase in any of the cytokines. These results should be treated with caution, as there was only enough sample material to run the tests twice.

**2007: Wear Particles Generated from Studded Tires and Pavement Induces Inflammatory Reactions in Mouse Macrophage Cells** [67].

During this study, mouse macrophage cells were used to assess the inflammatory effects of studded tyre and pavement wear in vitro, compared to urban street and subway station particles. The tyre and pavement wear particle samples were collected from the VTI road simulator using studded tyres and two types of pavements: dense asphalt with granite and stone mastic asphalt with quartzite, which means that there cannot be a direct comparison between granite and quartzite.

The results showed that all the particle types studied were able to induce cytokine release, with subway particles being the most potent for TNF-α and street particles for IL-6. In both cases, granite caused a release of 2–3 magnitudes higher than quartzite, which is a similar result to a previous study on human cells. As the deferoxamine mesylate (DFX) inhibitor had no inhibiting effect on granite or quartzite, it is possible that the TNF-α increase in not caused by the iron content or radical hydrogen generation of these particles, while it might be for street and subway particles. Only granite and street particles induced NO production, while quartzite did not, which is probably due to a difference in the mineral composition between the two pavement tyres, as the same tyres were used for both samples. While only subway particles caused an arachidonic acid release, all samples were able to induce lipid peroxidation and ROS formation, around the same magnitude for both granite and quartzite pavement.

**2007: Organic Extract of Tire Debris Causes Localized Damage in the Plasma Membrane of Human Lung Epithelial Cells** [63].

In this paper, the toxicity of tyre debris organic extracts on A549 human alveolar epithelial cells was studied in vitro, through a series of experiments with different exposure times and doses, assessing any modifications in the plasma membrane molecular composition and lipid microdomain expressions. The tyre samples were collected by rotating a tyre against a steel brush. The organic extracts were then extracted with a Soxhlet apparatus, and three samples with doses of 10, 60, and 75 μg/mL were exposed to the cell cultures for 24, 48, and 72 h.

Trypan Blue and MTT assays were used to assess cytotoxicity. The percentage of Trypan Blue-positive cells increased in a dose-dependent manner but remained unaffected by time, although it always remained under 5%. There was also a time- and dose-dependent increase in lactate dehydrogenase (LDH) in the medium after 48 h, which is an index of necrosis. There was no significant difference in cell proteins or in the membrane-enriched fraction (MEF) cholesterol content between the groups. A significant increase in lipid microdomains was noted, more specifically, dose-dependent for flottilin-1 (FLOT-1), time- and dose-dependent for caveolin-1 (CAV-1), and time- and slightly dose-dependent for lipid raft marker CD55. There was a 20-fold increase in heme oxygenase-1 (HO-1) content in the detergent-resistant membrane (DRM) after exposure from 24 to 72 h. Lastly, an increase in invaginations was observed; however, other organelles did not present a modified morphology.

**2008: Organic Compounds in Tire Particle Induce Reactive Oxygen Species and Heat-Shock Proteins in the Human Alveolar Cell Line A549** [91].

During this in vitro study, tyre particle organic extracts were used to assess their toxicity (ROS production and heat shock protein 70 (Hsp70) expression) to human lung cells. The tyre samples were collected by milling a new vehicle tyre with liquid nitrogen (cryo-fracturing), and then the organic extract was extracted using a Soxhlet apparatus. The authors note that the morphology and chemical composition of the tyre debris particles created in the laboratory are very similar to those of particles collected in the environment.

For both parameters studied, the exposure doses used were 10, 50, 60, and 75 μg/mL, and for the Hsp70 expression measurements, the cells went through 24, 48, and 72 h of treatment. The study concluded that the earliest observable cell response to tyre debris organic extracts is ROS production, which was assessed through fluorescent probe 2′7′-dichlorodihydrofluorescein diacetate (DCFH-DA). A statistically significant dose-dependent increase in fluorescence was observed after 2 h of exposure with 50 (+82%), 60 (+99%), and 75 μg/mL (+127%). When testing the expression of Hsp70, there was a much more significant expression at 72 h and lower doses compared to 24 and 48 h. This suggests that Hsp70 might play a role in protecting alveolar cells against damage caused by particles.

**2008: Properties and Toxicological Effects of Particles from the Interaction Between Tyres, Road Pavement and Winter Traction Material** [68].

In this paper, the toxicological effects of different traffic-related particles were studied in vitro using human monocytes. Seven different combinations of tyres and pavements (studded tyres, non-studded winter tyres, dense asphalt concrete with granite, stone mastic asphalt with quartzite, no traction material, crushed stone as traction material, and natural sand as traction material) were used at different speeds in the VTI lab to collect the particles.

All the samples tested induced the cytokine secretion of TNF-α, IL-6, and IL-8; however, the granite pavement and street particles consistently induced higher secretion compared to the quartzite pavement and subway particles. Only street particles were affected by the inhibitors tested. Interestingly, the granite samples induced a TNF-α increase for all doses except for doses between 100 and 250 μg/mL, whereas quartzite induced an increase for all doses except for 100 μg/mL. Granite and quartzite both induced an increase in IL-8 only between 10 and 50 μg/mL and then a decrease for higher doses, while granite induced a higher secretion of IL-6 at 50 and 100 μg/mL compared to quartzite.

**2008: Mechanisms Related to the Genotoxicity of Particles in the Subway and from Other Sources** [60].

This study assessed the genotoxicity of subway particles compared to other sources, like streets, diesel, tyre–road, etc., by testing human lung epithelial cells in vitro. Samples of tyre wear particles were collected from the VTI road simulator (no further details were mentioned) using studded tyres and asphalt concrete (ABT) pavement; however, after analysis, it was established that the samples contained mostly stone material from the pavement.

All the samples tested caused a significant increase in cells with mitochondrial depolarisation; however, the tyre–road sample caused the least amount of damage with only 37% cells with depolarised mitochondria, compared to diesel exhaust particles which caused the highest amount of damage with 73%. The authors chose to assess mitochondrial depolarisation because of its biological significance, as mitochondrial damage has important known biological effects, including the initiation of apoptosis or cell death.

Subway particles were the only sample able to cause a significant increase in ROS compared to the control and caused a much higher response compared to the tyre–road sample (*p* < 0.05). Other parameters were also tested; however, tyre–road particles were not included.

**2008: Cardiopulmonary Responses of Intratracheally Instilled Tire Particles and Constituent Metal Components** [43].

In this paper, two tyre samples were used to assess their cardiopulmonary toxicity in mice, through an intratracheal installation. The tyre samples used were collected from a recycled tyre material (styrene butadiene rubber) and from two tyre scraps and were ground to the appropriate size.

The bronchoalveolar lavage fluid (BALF) analysis showed a significant increase in the number of total lavageable cells and neutrophilic influx; however, the increase was much greater in the tyre scrap sample compared to the recycled tyre material. The scrap sample also caused a significant increase in γ -glutamyl transpeptidase (GGT), LDH, and β-N-acetylglucosaminidase (NAG) after 24 h.

After the metal analysis, it was evident that the only two elements that could be correlated to the changes in toxicity between the two samples were Zn and Cu. Because of this, the effect of these two metals was assessed further by executing exposure experiments with a Zn sample, a Cu sample, and a combination of Zn and Cu. At 4 h, Cu and Cu + Zn caused an increase in total lavageable cells, whereas at 24 h, Zn and Cu + Zn caused an increase in total lavageable cells. In both cases, the combined sample caused a much greater effect than the individual ones. All the samples tested also increased the levels of BALF lung injury markers and decreased the mitochondrial aconitase activity.

**2009: Lung Toxicity Induced by Intratracheal Instillation of Size-Fractionated Tire Particles** [44].

In this study, the size dependency of the adverse health effects of tyre particles was assessed in mice in vivo through the intratracheal installation of particles. The intratracheal installation was chosen as the exposure method in order to facilitate comparison with other studies on similar animal models and because it offers easy delivery to the lungs; however, its main disadvantage is that there is often uneven distribution within the alveolar region because of preferential deposition in the major conducting airways. The tyre wear particle samples were collected from tyre scraps from a commercial tyre recycling plant and then milled with liquid hydrogen in order to achieve the right particle size. The particle samples were then size-separated by air elutriation into PM_2.5_ and PM_10_. The exposure samples used for the installations contained 10, 100, and 200 μg of PM_2.5_ or PM_10_ particles in 100 μL of isotonic saline solution with 0.01% Tween 20.

After 24 h, the bronchoalveolar lavage fluid (BALF) was analysed. The total cell count showed no dose–response relationship and was only increased in the middle dose. Both size fractions induced significant granulocyte infiltration, shown by the increase in the polymorphonuclear neutrophilic leukocyte (PMN) percentage and decrease in macrophage percentage, which was dose-dependent for PM_10_ but not PM_2.5_. No statistical difference was observed in the lymphocyte percentage for PM_10_, and only the lowest dose of PM_2.5_ showed a significant increase. A significant increase in the total protein and alkaline phosphatase was observed for PM_2.5_ but not for PM_10_, and an increase in LDH activity was observed for the two highest doses of PM_2.5_ and the highest dose of PM_10_. No modification of β-glucuronidase was found for PM_2.5_, although the enzyme was elevated for the highest dose of PM_10_. An increase in GSH (glutathione) was observed at the highest dose of PM_2.5_, but no significant difference was found for PM_10_, and no significant difference in superoxide dismutase (SOD) activity was observed. Almost no pro-inflammatory cytokine release (macrophage inflammatory protein-2 (MIP-2) and TNF-α) was detectable, possibly due to the affinity of cytokines to adsorb to TPs.

The histopathological study for PM_10_ showed intact respiratory parenchyma, with particle aggregates lining the alveolar walls and numerous macrophages engulfing PMs. The highest doses showed heavy inflammatory and degenerative processes. For PM_2.5_, finely dispersed particles were observed in alveolar spaces, with fewer particle aggregates compared to PM_10_. PM_10_-instilled mice showed macrophage-mediated clearance and inflammatory tissue infiltration, whereas PM_2.5_-instilled mice showed congested respiratory tissue with haemorrhagic and necrotic phenomena.

**2010: Comparative Acute Lung Inflammation Induced by Atmospheric PM and Size-Fractionated Tire Particles** [45].

In this study, the effects of size-fractioned tyre particles and atmospheric PMs were studied in vivo through intratracheal instillation exposure in mice with a 3 h recovery period, while previous research looked at a 24 h recovery period. The tyre particle samples were collected from tyre scraps from a tyre recycling plant and then milled with liquid hydrogen in order to achieve the right particle size, whereas the atmospheric PM samples were collected in Milan in 2007.

The BALF was studied to assess cytotoxicity. A statistically significant PMN increase was only observed for 2.5 μm tyre particles (TP_2.5_) and PM_2.5_, suggesting that the finer particles induce the highest cellular inflammatory response. Macrophage inflammatory protein-2 (MIP-2) and TNF-α showed a significant increase due to both TPs and PM; however, TNF-α was mostly increased significantly after TP_2.5_ and TP_10_ exposure. The casp8 increase was four times higher in TP_2.5_-treated mice compared to controls. No significant increase in NF-kB p50 was observed. Heme oxygenase-1 had a strong increase after TP_2.5_ and PM treatment. The lung histology showed that, contrary to PM, TPs were present in the airway and alveolar spaces lining the epithelial tissues. TP_10_-instilled mice presented large particle deposits, whereas TP_2.5_ deeply penetrated the alveolar sacs, engulfing macrophages and adhering to the alveolar epithelial cells, sometimes accompanied by diffuse necrotic changes to the alveolar walls.

Overall, the TP_2.5_ samples reached the alveolar spaces and produced an acute inflammatory response, whereas TP_10_ particles reached mostly the bronchial spaces and did not cause a significant response. On the other hand, PM_2.5_ induced less of an inflammatory response compared to PM_10_, which shows that the inflammatory mechanism is different for atmospheric PM and for tyre particles.

**2011: Wear Particles from Studded Tires and Granite Pavement Induce Pro-inflammatory Alterations in Human Monocyte-Derived Macrophages: A Proteomic Study** [92].

During this proteomic study, the effects of tyre wear particles on human macrophages were analysed in vitro. The chosen set of cells was monocyte-derived macrophages (MDMs), which are known to resemble human alveolar macrophages both morphologically and functionally. The tyre wear particles were collected from the VTI road simulator from studded tyres on dense asphalt with granite at 70 km/h. In total, 626 proteins were detected and compared for intensity for both the tyre-wear-particle-exposed cells and endotoxin-exposed cells. Of those, 119 were identified by time-of-flight mass spectrometry (TOF-MS) and studied.

Seven of the proteins investigated significantly decreased, whereas three significantly increased after tyre wear particle exposure. More specifically, proteins involved in inflammatory responses, like markers of inflammation and fibrosis, were up-regulated, whereas proteins involved in cellular functions, like energy metabolism and protein synthesis, were down-regulated. After endotoxin exposure, four proteins were down-regulated, of which two were also down-regulated after wear particle exposure and two were not. The two proteins that were up-regulated after wear particle exposure were also up-regulated after endotoxin exposure. Since endotoxin exposure resulted in a similar, but not completely identical, response, it can be hypothesised that there are other factors affecting the toxicity of wear particles.

**2012: Evaluation of Potential for Toxicity from Subacute Inhalation of Tire and Road Wear Particles in Rats** [57].

In this paper, the lung toxicity of tyre and road wear particles was assessed through an in vivo inhalation study on mice. The particle samples were collected in a road simulator laboratory with an interior drum testing system with asphalt pavement, from one summer and one winter silica-based tyre, one carbon-black-based summer tyre, and a combination of them.

The results showed no signs of general toxicity, such as dead animals, abnormal breathing, or abnormal behaviour. The lavage fluid analysis showed an unchanged cell differential profile with unchanged markers for cytotoxicity and inflammatory cytokines. The lung tissue analysis showed normal values for HO-1 and thiobarbituric acid reactive substances (TBARSs), showing a low potential for inducing oxidative stress. Lastly, the histopathological analysis showed, at higher doses, few widely scattered minimal focal areas of subacute inflammatory cell infiltration of mononuclear cells in the alveolar wall and even fewer in the alveolar spaces. The results were consistent with a previous intratracheal instillation study which evaluated similar endpoints.

**2019: Exposure to Particle Debris Generated from Passenger and Truck Tires Induces Different Genotoxicity and Inflammatory Responses in the RAW 264.7 Cell Line** [93].

In this study, the cyto- and genotoxic effects of passenger and truck tyre particles on mice macrophages were assessed in vitro. The samples were created by cryo-fracturing both light- and heavy-duty tyres.

The results showed that at 4 h there was a slight reduction in cell viability in passenger tyres at 25 μg/mL and in truck tyres at 100 μg/mL. There was, however, a decrease in metabolic activity at 24 h, followed by a significant increase in metabolic activity at 48 h, for both samples tested. The authors speculate that the observed trend in viability may be due to the cells first absorbing and then acting as macrophages. For the CBPI index, there was an evident increase in all the conditions tested using passenger tyres and only a slight increase at 10 μg/mL using truck tyres. There was also a significant increase in the formation of micronuclei at all concentrations for both samples.

At 4 h of incubation, there was a slight increase in TNF-α for both samples, and after 24 h, there was a significant increase in the release of TNF-α. Passenger tyre samples caused a much greater release of pro-inflammatory cytokines, whereas truck tyre samples showed a low and steady early release. During all the experiments, there was no dose-dependent response.

**2020: A Novel 3D Intestine Barrier Model to Study the Immune Response Upon Exposure to Microplastics** [61].

In this study, a novel, three-dimensional in vitro intestinal model was used in order to study the effects of ingested microplastics on human health. The microplastic samples were created through cryo-milling polymer pellets and truck tyre materials. The 3D model was created with monocyte-derived macrophages (MDMs) and monocyte-derived dendritic cells (MDDCs) at a 1:4 ratio in the lower layer and epithelial Caco-2 cells and mucus secreting goblet cells HT29-MTX at a 9:1 ratio in the upper layer.

The results showed no significant cytotoxicity, as the cell viability did not change significantly after 48 h. There was also no significant release of pro-inflammatory cytokines for all exposure times when testing IL-8, TNF-α, and IL-1β. Lastly, there was no change in the barrier integrity of the co-cultures.

**2021: In-Vitro Assessment Reveals the Effects of Environmentally Persistent Free Radicals on the Toxicity of Photoaged Tire Wear Particles** [94].

In this study, photoaged tyre wear particles, and hence environmentally persistent free radicals (EPFRs), were used in order to assess their toxicity through a series of in vitro experiments on macrophages derived from mice. The tyre particle samples were created by ball milling with liquid nitrogen tyre pieces from a tyre recycling facility, in order to reach the correct size. The artificial aging of the particles was carried out under a simulated solar light from a photoreactor with a xenon lamp.

The results showed a decrease in cell viability to 73.5% for cells exposed to 10-day photoaged particles, whereas cells exposed to 30- and 60-day photoaged particles had even lower cell viability (54.9%). Exposure to photoaged particles was able to induce ROS generation, which also increased with a longer irradiation time of the samples. The results showed that the particles also caused an increase in the mRNA levels of inflammatory factors, like IL-6, TNF-α, and iNOS, which increased for longer photoaged samples. After the use of a free radical scavenger, the cell viability increased to 66–90%, and the increase in ROS generation and mRNA levels decreased, suggesting that the generated free radicals contribute to the particles’ cytotoxicity, as there was a clear increase in EPFRs with longer irradiation times.

**2022: Mutagenicity of PM_10_-bound PAHs from Non-Exhaust Sources** [62].

In this paper, the mutagenicity of PM_10_-bound PAHs was analysed in vitro using Salmonella typhimurium bacteria (Ames test). The road dust samples were collected from the VTI road simulator laboratory with a real-life representative method for two different summer tyres and using in situ resuspension chambers in two Portuguese cities, and then the organic compounds (PAHs) were extracted.

It was concluded that the particles did not show any mutagenic response at the concentrations used, with and without metabolic activation. This could be due to the small amounts of samples used or due to the fact that only two strains of bacteria were used, even though, according to the OECD guidelines, at least five strains should be used.

**2022: Inhaled Tire-Wear Microplastic Particles Induced Pulmonary Fibrotic Injury via Epithelial Cytoskeleton Rearrangement** [95].

In this study, the pulmonary effects of exposure to tyre wear microplastic particles were assessed through in vivo experiments on mice, as well as in vitro experiments on human bronchial epithelial BEAS-2B cells. The samples were collected through grinding and ball milling a tyre.

The study showed that exposure to particles caused restricted ventilatory dysfunction and a fibrotic pathological response in the lungs. The peak expiratory flow (PEF) parameter, which reflects the inhalation capacity of the lungs and the functional status of the large airways, was decreased after exposure, while the tissue damping and tissue elastance values were elevated, which is consistent with pulmonary fibrosis. The toxicological evaluation showed that the proportion of macrophages decreased by 20% and the proportion of lymphocytes increased by 22% in the high-exposure-dose group.

Moreover, the lung function parameters showed histopathological changes in the lungs consistent with pulmonary fibrosis. Major histopathological changes were observed, including the congestion of capillaries, alveolar stenosis, alveolar wall thickening, etc. Significant collagen deposition was observed after Sirius Red staining of the lung tissues, as well as black particles in the alveolar lavage fluid macrophages and alveolar cavities.

The miR-1a-3p genome was significantly down-regulated in the mice lung tissues during in vivo experiments, as well as during in vitro experiments. However, the messenger ribonucleic acid (mRNA) and protein levels of the target gene twinfilin-1 both increased after exposure, and the cytoskeleton exhibited a fractured and sparse appearance with the skeleton network and actin-stress fibres missing. This shows that twinfilin-1, which is regulated by miR-1a-3p, played an important role in the cytoskeleton rearrangement and cell migration, caused by tyre wear particle exposure.

**2023: Human Airway Organoids as 3D In-Vitro Models for a Toxicity Assessment of Emerging Inhaled Pollutants: Tire Wear Particles** [69].

In this paper, an in vitro study was conducted using 3D human airway organoids (hAOs) to assess the toxicological effect of tyre wear particles on human airways. To create the 3D hAO model, primary human bronchial epithelial cells were used. The samples were collected through grinding and ball milling a tyre.

The samples were able to induce significant cytotoxicity at concentrations higher than 200 μg/mL and significant ROS generation in a dose-dependent manner, as well as an increase in oxidative stress-related and cytokine-related gene expressions, including TNF-α, IL-6, and ccl2. The particles were found to be clustered around the epithelial cells, and smaller particles were found inside the cells. The radial size of the hAOs exposed to a concentration of 100 μg/mL of particles was smaller than that of the control. The exposure also caused a significant increase in early and late apoptotic cells, with the late apoptotic cells mainly concentrated in the interior of the hAOs and the early ones in the periphery.

**2023: Particle Debris Generated from Passenger Tires Induces Morphological and Gene Expression Alterations in the Macrophages Cell Line RAW 264.7** [64].

In this in vitro study, particles generated from tyre debris were used to assess their toxicity by exposing mice cells to the particles. The samples were collected from a road simulator device and then cryo-milled in order to obtain smaller particles, which is not representative of tyre wear.

The proliferation study only showed a significant response after prolonged stress and high concentrations, which is in line with chronic toxicity instead of acute. The comet assay showed no increase in the tail DNA %, tail moment, or olive tail moment, indicating no significant DNA damage. The gene expression of p53 and p21 increased significantly at 48 h of exposure; however, while p53 increased with both concentrations tested, p21 only increased at the higher concentration. An increase in transcripts of the proteins involved in apoptosis was observed after 48 h. The immunoblot analysis was consistent with the cells’ proliferation, indicating that only prolonged stress can induce a complex toxicological response.

**2023: Oral Exposure to Tire Rubber-Derived Contaminant 6PPD and 6PPD Quinone Induce Hepatotoxicity in Mice** [70].

In this paper, the health effects of 6PPD and 6PPDQ on the liver of mice was assessed in vivo by oral exposure for 6 weeks. The results showed changes in the liver weight and aspartate aminotransferase/alanine aminotransferase (AST/ALT) ratios at higher concentrations, suggesting liver dysfunction, even though haematoxylin and eosin staining showed no serious liver damage. Oil staining revealed severe lipid accumulation in the liver in a dose-dependent manner. Higher exposures caused a higher level of serum triglycerides and pyruvate in the mice. The relative mRNA level of pyruvate kinase decreased after 6PPDQ exposure, while the nRNA levels of glucose kinase did not change significantly.

6PPDQ was found to cause obvious inflammation in the liver, which increased the NOD-1, TNF-α, IL-6, and IL-22 nRNA levels. Lastly, the bioresidues of both compounds increased in the liver in a dose-dependent manner.

**2023: Four-Week Repeated Exposure to Tire-Derived 6PPD Quinone Causes Multiple Organ Injury in Male BALB/c Mice** [71].

In this study, the toxicity of 6PPDQ in mice was assessed in vivo by single intraperitoneal injections and repeated intraperitoneal injections. Both single and repeated exposures did not cause any changes in body weight and food intake in mice. The results showed that in the single-injected mice, there were no significant changes in the organ indexes and biochemical parameters, and no pathological changes were observed.

On the other hand, repeated injections were able to cause an increase in the organ indexes for the liver, kidney, lung, testis, and brain and a decrease in the spleen index. Alanine aminotransferase (ALT), aspartate aminotransferase (AST), alkaline phosphatase (ALP), urea, and creatinine were all increased after repeated exposure, while they remained unchanged after single exposure. Significant pathological changes occurred in the liver, kidney, lung, spleen, testis, and brain. The biochemical parameters for the liver and kidney were significantly up-regulated after repeated exposure, and 6PPDQ was accumulated in the liver and lungs of mice.

**2024: Exposure of RAW264.7 Macrophages to Exhaust Emissions (Gases And PAH) and Non-Exhaust Emissions (Tire Particles) Induces Additive or Synergistic TNF-A Production Depending on the Tire Particle Size** [96].

In this paper, the toxicity of combined exhaust gases and benzo[a]pyrene (representative of PAHs) and tyre particles in mice cell macrophages was assessed in vitro. The cell line was exposed to tyre particles of different sizes (6–113 μm), either alone or in combination with benzo[a]pyrene (B[a]P) and a mixture of exhaust gases (CO_2_, CO, NO, NO_2_). The tyre particles used were produced by cryo-grinding pieces of tyre tread from commercial tyres with a maximum wear rate of 40%. The tyre particles were separated in fractions of 70 μm, 30 μm, 15 μm, and 5 μm.

After 24 h of exposure, no cytotoxicity was induced, irrespective of the condition of exposure of cells, and after both 90 min and 24 h of exposure, there was no significant ROS production, indicating that there was no oxidative stress. However, when assessing the production of the TNF-α proinflammatory cytokine, it was evident that B[a]P was able to cause an inflammatory response, and tyre particles caused a size-dependent response, while exhaust gases did not. More interestingly, when assessing the combined exposure, there were two different responses depending on the size of the tyre particles. For the smaller sizes, there was a slight increase in TNF-α production, suggesting that the combined effects were additive, and for the larger particles, there was a disproportionate increase in TNF-α production, suggesting a synergistic effect.

**2024: An In-Vitro Comparison of the Toxicological Profiles of Ground Tire Particles (TP) and Actual Tire and Road Wear Particles (TRWP) Emissions** [65].

In this study, the in vitro toxicity of ground TPs and actual TRWP emissions of similar size collected from road traffic was assessed by incubation with alveolar macrophages for 24 h and assessing the cytotoxicity, proinflammatory response, and oxidative stress. The TPs were created by the cryogenic grinding of a tyre, while the TRWPs were collected during the on-road testing of a moving vehicle in order to include both tyre and road wear particles, as well as resuspended particles.

The results showed that neither of the particles tested induced cytotoxicity or oxidative stress; however, they were both able to induce a concentration-dependent proinflammatory response. The TNF-α production was slightly higher for the TRWP samples, at all concentrations tested.

#### Appendix A.2.2. Aquatic

**2005: Toxicity of Tire Debris Leachates** [56].

During this study, the toxicity of tyre debris leachates was studied in vitro and in vivo by exposing different aquatic organisms to tyre debris eluates produced in different ways. The tyre samples were collected in a laboratory, by rotating a new tyre against a steel brush, which is not representative of normal tyre wear.

The results for *R. subcapitata* showed a strong correlation between leachate concentration and algal growth inhibition. After testing with the addition of ethylene diammine tetra acetic acid (EDTA), the toxicity was reduced, indicating the presence of a potential organic toxicant. When testing *D. magna* for acute and chronic toxicity, a correlation between the death rate and concentrations was evident, and after five days, all juveniles were dead. During the frog embryo teratogenesis assay—Xenopus (FETAX) test, it was seen that for concentrations up to 50%, leachates were not embryolethal to *X. laevis*; however, at a 100% concentration, there was an 80.2% mortality percentage for 50 g/L, and only 26.8% for 100 g/L, which shows that the 50 g/L exposure caused a much stronger toxicological response than 100 g/L. This difference in toxicity was also evident in the malformed larva percentages and teratogenicity.

The authors speculate that while many factors play a role in the toxicity of the leachates, like the pH, particle size, and Zn concentration, one of the main reasons why 50 g/L produced a stronger response is that the particles aggregate, creating a smaller surface area and hence less opportunity to interact with the organism and cause toxicity.

**2007: Tire Debris Organic Extract Affects Xenopus Development** [97].

In this paper, the embryotoxic effects of tyre debris organic extracts on Xenopus (frogs) were assessed, through a series of experiments, including the frog embryo teratogenesis assay—Xenopus (FETAX). The tyre debris samples were created in a laboratory by cryo-fracturing tyre scraps.

The results showed that the non-observable effect concentration (NOEC) was 50 mg/L, and the mortality and percentage of malformed larvae were significantly higher than the control at 80 mg/L; however, a good concentration response was only observed in the percentage of malformed larvae. The probit analysis gave a 144.6 mg/L TC50. At 120 and 140 mg/L, many of the subjects were plurimalformed. The histological screening showed mostly ocular malformations, as well as severe vacuolisation and necrosis in the liver and axial musculature.

**2009: Impacts of Weathered Tire Debris on the Development of *Rana sylvatica* Larvae** [98].

In this study, the toxicological effects of tyre debris on frogs (*R. sylvatica*) were assessed by exposing eggs and larvae to aged sediments containing tyre debris (Zn^2+^) or ZnCl_2_, through metamorphosis. The tyre debris samples were collected from a scrap tyre facility.

The experiments showed that zinc was able to leach from the tyre debris through the aging process and that the organisms exposed to tyre debris and ZnCl_2_ has significantly higher tissue zinc accumulation. Exposure to both ZnCl_2_ and tyre debris was able to cause an increase in the time for larvae to complete metamorphosis, from 46 days to 52.8 and 52.5 days, respectively. Another observation was that the increased metamorphosis time caused a decrease in the subjects’ mass.

**2009: Toxicity Assessment of Sequential Leachates of Tire Powder Using a Battery of Toxicity Tests and Toxicity Identification Evaluations** [99].

In this study, the toxicity of tyre leachates was assessed in vivo and in vitro through a series of experiments on green algae, crustaceans, and zebra fish eggs. The samples used were collected by abrading three tyres (one slightly worn, two heavily worn; all different brands) using a rasp and then by adding the tyre powder to purified water multiple times, in order to retrieve the sequential leachates. The zebra fish eggs did not present any consistent toxicity for the concentrations used and hence were excluded from further analysis.

The slightly worn tyre was shown to cause the most significant toxic response, although, as the three tyres were of different types and brands, its toxicity cannot be attributed to the fact that the tyre was not very worn. The sequential leachates were also shown to be much less toxic than the first one.

The Friedman test showed that the toxicity was likely due to both metal toxicants and lipophilic organic compounds. According to the authors, zinc is the metal causing the most toxicity because of the relative sensitivity exhibited. However, the importance of metals and organic compounds in toxicology seems to vary significantly, which the authors speculate might be due to the used tyres leaching more zinc and less organic compounds compared to new tyres.

**2010: Toxicity of Tire Wear Particle Leachate to the Marine Macroalga, *Ulva lactuca*** [100].

In this paper, the toxicity of tyre wear particle leachates in the marine macroalga *U. lactuca* was assessed in vivo, as well as the release of Zn from the particles. The samples were created by abrading 20 end-of-life car tyres with a stainless steel file. The leachates were created using filtered sea water.

The results showed that exposure to increasing concentrations of leachate resulted in a non-linear reduction in the efficiency of photochemical energy conversion and an increase in Zn accumulation, in all except from the undiluted leachate. The phototoxicity was significantly lower when the Zn was added as Zn(NO_3_)_2_, suggesting that the organic components of the leachates are the main cause of the overall toxicity.

**2011: Acute Aquatic Toxicity of Tire and Road Wear Particles to Alga, Daphnid, and Fish** [101].

In this study, the acute toxicity of tyre and road wear particles was assessed in three different aquatic species in vivo through exposure to a sediment elutriate with different concentrations. The samples were collected from a road simulator laboratory in an interior drum testing system containing actual asphalt pavement in cassettes, using summer and winter silica-based tyres and one carbon-black-based summer tyre and combing the particles. In this experiment, three different aquatic species were assessed: *P. subcapitata* (algae), *D. manga* (crustacean), and *P. promelas* (fish).

Leachates that were prepared by incubating particles in water at 44 °C, both with and without sediment presence, were found to be acutely toxic to *D. manga* and to cause a concentration-dependent decrease in the survival rate. On the other hand, the leachates prepared at room temperature did not cause any toxicity, with or without sediment presence. The spiked sediment elutriates exhibited no acute toxicity in any of the organisms tested, and no concentration-dependent responses were observed. The toxicity identification evaluation was in agreement with the previous results. Toxicity identification evaluation (TIE) studies and chemical analysis showed zinc and aniline as possible candidate toxicants.

**2013: Chronic Toxicity of Tire and Road Wear Particles to Water- and Sediment-Dwelling Organisms** [102].

In this study, the chronic toxicity of tyre and road wear particles (TRWPs) (sediments and elutriates) was studied in vivo by exposing four different aquatic species. The samples were collected from a road simulator laboratory in an interior drum testing system containing actual asphalt pavement in cassettes, using summer and winter silica-based tyres and one carbon-black-based summer tyre and combing the particles.

The sediments spiked with TRWPs caused mild growth inhibition in Chironomus dilutes but had no effect on survival, growth, or reproduction in *Hyalella azteca*. On the other hand, the TRWP elutriates caused a slightly diminished survival in larval *Pimephales promelas* but had no effect on any of the endpoints tested in *Ceriodaphnia dubia*.

**2018: Ingestion and Chronic Effects of Car Tire Tread Particles on Freshwater Benthic Macroinvertebrates** [73].

In this paper, four freshwater benthic macroinvertebrates were exposed to tyre tread particles for 28 days to assess their toxicity to aquatic life. The samples were created through cryo-fracturing, which is not a representative method.

The results showed no significant relationship between the increasing concentration of tyre particles and the survival, growth, and feeding rate of *G. pulex* and *A. aquaticus* and the survival and growth of *Tubifex* spp., as well as the number of worms and growth of *L. variegatus*. Furthermore, the chemical analysis showed that only a small fraction of zinc was bioavailable, even though the tyre particles had a high intrinsic zinc content.

**2019: Acute and Long-Term Toxicity of Micronized Car Tire Wear Particles to *Hyalella azteca*** [103].

In this study, the toxicity of tyre wear particles and tyre leachates in *Hyalella azteca* was assessed after ingestion. The tyre samples were collected by grinding a road-worn tyre against a course grindstone. *H. azteca* was shown to indiscriminately ingest tyre wear particles with a gut residence time of between 24 and 48 h.

Tyre wear particles showed a typical concentration–response pattern; however, leachates did not. At low concentrations, the leachates appeared more toxic, whereas at higher concentrations, the particles seemed to cause a stronger response. The toxicological profiles were different, suggesting a dissimilar mechanism of toxicity between particles and leachates. Mortality, reproductive output, and net growth were significantly affected at higher concentrations after 21-day exposure.

**2020: Chemical Composition and Ecotoxicity of Plastic and Car Tire Rubber Leachates to Aquatic Organisms** [83].

In this study, the ecotoxicology of certain microplastics, including tyre particles, was assessed in vitro through a series of experiments on two microalgae (*R. subcapitata* and *S. costatum*) and one mussel (*M. galloprovincialis*) species. The tyre samples were taken from tyre-derived granulate reference materials.

After chemical analysis, it was found that tyres and polyvinyl chloride (PVC) had the highest content of tentatively identified organic additives, and tyre particles and leachates were found to contain benzothiazole, cobalt, and zinc as possible toxicants. Benzothiazole was not present in any other samples. Tyre leachates were able to lower the pH in both the marine and freshwater samples.

Leachate toxicities towards algae varied significantly between the different samples tested and between the two different algal species. All leachates inhibited algal growth, with tyre and PVC leachates having the biggest effect, which could be directly linked to the higher chemical additives present, particularly Zn. When assessing lysosomal membrane stability (LMS), which is a known biomarker of cellular and general stress, tyre leachates were also found to be the most toxic for marine mussels, exhibiting significant reductions with respect to the control. Tyre and PVC leachates displayed a significant down-regulation of gamete fertilisation, with a strong dose-responsiveness. While all the leachates were able to induce a significant inhibition of normal embryonic development, the tyre leachate samples showed a strong reduction. Lastly, tyre leachates caused a significant reduction in larvae motility (48 h) and survival (144 h).

**2020: Increased Temperature and Turbulence Alter the Effects of Leachates from Tire Particles on Fathead Minnow (*Pimephales promelas*)** [77].

In this study, two samples with tyre particles that had been exposed to four different levels of each physical stressor (temperature, mechanical stress, UV, and CO_2_) were used in order to assess their toxicity to *Pimephales promelas*. The samples were manually cut into small pieces from two new tyres, which is not a representative method.

In the temperature experiments, there was no significant effect on the time to hatch; however, hatching success decreased in the Tyre 2 exposures. Significant differences in heart rates were observed for different materials and different temperatures. Increasing deformities and shorter lengths were observed with increasing temperature, although Tyre 2 caused more severe deformities.

Both mechanical stress and UV exposure caused very similar effects to temperature changes, where the effects were consistent between the two tyre types, although Tyre 2 caused more severe effects. Although carbon dioxide exposure can change the water pH levels, the effects were not significant enough to influence the leaching process. The effects were similar to the previous experiments.

All the embryos used in the experiments had a noticeable lack of pigmentation in their eyes, despite the treatment. The authors suggest that this loss of pigmentation is due to the exposure to PAHs, Zn, and other possible toxicants found in the tyre leachates.

**2020: Toxicological effects of micronized tire crumb rubber on mummichog (*Fundulus heteroclitus*) and fathead minnow (*Pimephales promelas*)** [104].

In this study, the aquatic toxicity of crumb rubber was assessed in vivo in *F. heteroclitus* and *P. promelas*. The two fish species were exposed to different concentrations with a 7-day static renewal exposure. The crumb rubber samples used in this study were created from raw materials derived from passenger and truck tyres, using a wet grinding technology.

The results revealed that the highest concentration tested (6 g/L), which is close to the assumed LC50, caused partial mortality in *P. promelas*. The bile fluorescence and EROD assay indicated that there was a presence of PAHs and that four-ring PAH compounds were the most bioavailable.

**2021: Tire Wear Particle and Leachate Exposures from a Pristine and Road-Worn Tire to *Hyalella azteca*: Comparison of Chemical Content and Biological Effects** [105].

In this study, *Hyalella azteca* was exposed to tyre wear particles and leachates from new and worn tyres in order to assess their toxicity in aquatic life. The tyre particle samples were created by grinding two tyres.

In the acute toxicity tests, the pristine tyre sample caused greater toxicity compared to the used tyre samples, for both the suspensions and the leachates. However, in the road-worn tyre samples, the leachates caused higher toxicity at low concentrations, whereas the particle suspensions caused higher toxicity at higher concentrations. This is also shown by the NOEC and lowest observed effect concentration (LOEC) values, which are lower for the leachates. On the other hand, for the pristine tyre samples, the particles were shown to be equally or more toxic than the leachates, although this was not supported by the LOEC values.

When testing long-term toxicity, the tyre particle suspensions showed no significant effects on mortality and reproduction; however, growth was significantly reduced after pristine tyre exposure at the highest concentration tested.

**2021: Treading Water: Tire Wear Particle Leachate Recreates an Urban Runoff Mortality Syndrome in Coho but Not Chum Salmon** [76].

In this study, the toxicity of tyre wear particle leachates for coho and chum salmon was assessed and compared to roadway runoff toxicity. The tyre particle samples were generated by grinding nine unique tyres, which is not a representative method. High concentrations of 6PPDQ were present in the leachate.

The tyre leachate was acutely lethal to coho salmon at similar concentrations to roadway runoff, with similar behaviours and blood parameters impacted. On the other hand, chum salmon appeared to be insensitive to the leachate at concentrations lethal to coho salmon, the same as with roadway runoff. Coho salmon had 25–50% mortality at lower exposures and 100% mortality at intermediate and high exposures. In sublethal exposures, coho salmon seemed to be severely symptomatic and lethargic, whereas chum salmon displayed no behavioural changes. The results showed an increase in haematocrit and a decrease in plasma Na and pH in coho salmon, whereas chum salmon was unaffected by the exposure.

**2021: Altered Gene Expression in Chironomus Riparius (Insecta) in Response to Tire Rubber and Polystyrene Microplastics** [84].

In this study, twelve genes were assessed for alterations in an aquatic insect after polystyrene and tyre rubber microplastic exposure. The samples were obtained as a fine powder after cryo-grinding, which is not a representative method.

Overall, mortality was not affected by this short-term exposure; however, chronic exposure effects were not assessed. The results showed that several genes encoding for heat shock proteins were overexpressed, and genes coding for manganese superoxide dismutase and the FK506-binding protein were altered. One hsp gene and genes related to biotransformation and detoxification were not altered. Overall, the microplastics caused cellular stress which led to some gene alterations but did not cause any mortality.

**2022: PET Particles Raise Microbiological Concerns for Human Health While Tyre Wear Microplastic Particles Potentially Affect Ecosystem Services in Waters** [85].

This study compared the toxicological effect of polyethylene terephthalate (PET) particles compared to tyre wear particles on the pathobiome of a freshwater microbial community. A natural bacterial community from coastal surface waters of Lake Maggiore was used as a basal community and mixed with water from the effluent of a wastewater treatment plant from Verbania (at 80% lake water, 20% wastewater treatment plant water). The tyre samples were created by shredding a used tyre.

The results showed that there was a significant increase in total cells with an increased proportion of tyre wear particles, as well as a significant increase in the number of bacterial aggregates composed of more than 10 cells. Microphotos confirmed the presence of large biofilms covering the whole surface of the tyre wear microplastics, and although the PET bacterial abundances were much lower, the phenotypes were more diverse compared to the tyre wear particle biofilm, where most morphologies were small rods. At the end of the experiment, it was clear that the overall proportion of potential pathogens in each vessel was higher with higher proportions of tyre wear particles. Acinetobacter-belonging reads accounted for about 90% of the overall potential pathogenic reads when the tyre wear particles were predominant.

**2022: Internalization, Reduced Growth, and Behavioral Effects Following Exposure to Micro and Nano Tire Particles in Two Estuarine Indicator Species** [106].

In this study, the toxicity of tyre particles (TPs) and leachates was assessed in vivo by exposing *Menidia beryllina* and *Americamysis bahia* species to different concentrations of micro- and nanoparticles and leachates. The tyre samples were created by cryo-milling.

The results showed significant alterations in swimming behaviours for both species. In *A. bahia*, nano-TPs caused hyperactivity and 80% behavioural alterations, compared to 70% for micro-TPs. For both size fractions, the behavioural alterations were higher at increased salinities. Both *A. bahia* and *M. beryllina* larvae survival was not significantly decreased compared to the control. The behavioural alterations after leachate exposure in *M. beryllina* were significant; however, contrary to *A. bahia*, micro-TPs caused 79% behavioural alterations, and nano-TPs caused 75% alterations. For both species, the lowest salinity caused the highest variations when exposed to micro-TPs, whereas for the nano-TP and leachate exposure, the highest salinity caused the most behavioural changes.

In *A. bahia*, growth was significantly affected in a dose-dependent manner after micro-TP exposure for the two highest salinities but was not affected by nano-TP or leachate exposure. On the other hand, *M. beryllina* exhibited decreased growth with both nano-TPs and micro-TPs but not with leachate exposure. The results seem to suggest that ingestion is the most common interaction between these species and TPs, as there was concentration-dependent ingestion at all salinities.

**2022: Toxicity of Micro and Nano Tire Particles and Leachate for Model Freshwater Organisms** [107].

In this study, the toxicity of micro and nano tyre particles (TPs) was assessed in vivo using two model aquatic organisms and a crustacean. The samples were created through the cryo-milling of tyre pieces.

For all the samples tested, increasing concentrations significantly decreased the number of normal fish at 120 hpf in *D. rerio*. From the behavioural changes observed, the only one present in all exposures was a lack of spontaneous movement. The malformations and behavioural changes observed were similar for both nano-TPs and leachates. Certain abnormalities were only present after particle exposure and not after leachate exposure, namely axis malformations and hatching delays, as well as a significant increase in the total mortality.

All the samples tested caused mortality in *D. magna* after 48 h. In contrast to *D. rerio*, in *D. magna*, micro-TP exposure caused a higher toxicity than nano-TPs. Furthermore, *D. magna* was found to readily ingest the micro-TPs.

**2022: Toxicological Effects of 6PPD and 6PPD Quinone in Zebrafish Larvae** [108].

During this study, the toxicity of 6PPD and 6PPDQ was assessed in vivo in zebrafish larvae. The toxicity, morphology, swimming behaviour, heart rate, and oxygen consumption were all assessed after exposure.

Low concentrations of both compounds did not significantly affect hatching and the survival rate. However, higher concentrations significantly affected larval development. Many physical deformities were observed, including kyphosis, lordosis, and a reduction in eye size. A significant reduction in heartbeats per minute was observed, as well as a dose- and time-dependent effect on larval respiration. Moreover, a significant reduction in the size of the swim bladder was observed only in the 6PPD exposure groups.

Behaviour changes were also evident after exposure to both substances. Total distance travelled and locomotion were significantly decreased for both exposure groups. Furthermore, in the 6PPDQ group, there was a significant decrease in the velocity and an increase in the angular velocity of larvae. Overall, 6PPDQ was found to be toxic at lower concentrations compared to 6PPD; however, no significant mortality was observed.

**2022: Chronic Toxicity of Tire Crumb Rubber Particles to Mummichog (*Fundulus heteroclitus*) in Episodic Exposures** [109].

In this study, *Fundulus heteroclitus* was exposed to tyre wear particle leachates to assess their toxicity to aquatic life. The samples used were of crumb rubber.

CYP1A was up-regulated in the gills, intestine, and liver, although more prominent in the gills than in the intestinal tract, and the tissues receiving secondary antibody were all negative for CYP1A expression. There was a significant dose-dependent increased bile fluorescence, probably due to exposure to four-ring aromatic compounds (PAHs) like pyrene.

The data showed an increased plasma 8-OHdG concentration as a result of increased crumb rubber concentrations, which shows that there was DNA damage and repair due to cellular oxidative stress. Lastly, at high concentrations, there was a slight decrease in malondialdehyde (MDA) production, due to oxidative membrane damage, and an increase in the total GSH in the liver, as a result of chronic exposure.

**2022: Ecological Impact of End-of-Life-Tire (ELT)-Derived Rubbers: Acute and Chronic Effects at Organism and Population Levels** [110].

In this paper, the acute and chronic effects of end-of-life tyre-derived granules and powder on three freshwater organisms were assessed. Samples were collected from a tyre recycling treatment plant.

Acute tests on *D. magna* and *D. rerio* showed a 50% effect concentration (EC50) of over 100 mg/L for both the powder and granules. The authors then calculated a time-weighted arithmetic mean of Zn concentrations for *D. magna* and found an EC50 of over 16.2 μg/L of Zn for granule exposure and 43.5 μg/L of Zn for powder exposure. The same analysis was performed for *D. rerio*, and the results showed an EC50 of over 6.9 μg/L of Zn for granule exposure and 25 μg/L of Zn for powder exposure. This suggest that granules might cause a stronger response than powder samples. Lastly, a significant effect on the cell density of *P. subcapitata* was observed for both granule and powder exposure at 100 mg/L concentrations.

Chronic exposures showed a lowest observed effect concentration (LOEC) of 9.8 μg/L in *D. magna*, when testing for reproduction. The number of living offspring was also significantly impacted after exposure; however, there were no significant effects observed for all other parameters tested. In *D. rerio*, the LOEC obtained was 10 μg/L for survival, the juvenile fish weight parameter, and abnormal behaviour (only in powder exposure) and over 10 μg/L for hatching.

**2022: Effects of Polyester Fibers and Car Tire Particles on Freshwater Invertebrates** [86].

In this study, the ingestion capacity and effects of polyester fibres and car tyre particles were assessed in four freshwater invertebrates (*D. manga*, *H. azteca*, *A. aquaticus*, *L. variegatus*) for acute and chronic exposures. The samples, which were milled from end-of-life passenger tyres, were collected from a tyre granulate manufacturer (Genan).

The results showed that car tyre particle ingestion increased with increasing concentrations for all the species tested and was the highest in *D. manga*. There were no effects on mobility for *D. magna* after acute exposure; however, after chronic exposure, survival and reproduction were affected. There were significant differences between the effects on the two strains of *D. magna* used in the experiments.

The tests on *H. azteca*, *A. aquaticus*, and *L. variegatus* showed very low fibre ingestion but more significant tyre particle ingestion; however, there was no difference observed between day 4 and day 28, indicating that the particles did not accumulate within the organisms. Tyre particle uptake was much higher than fibre uptake, most likely due to the differences in shape and size. Ingestion was also found to be higher in water suspensions compared to particles mixed into the sediments. Lastly, there was no significant effect observed on survival after acute or chronic exposure for all three macroinvertebrate species.

**2022: Phenotypic Toxicity, Oxidative Response, and Transcriptomic Deregulation of the Rotifer *Brachionus plicatilis* Exposed to a Toxic Cocktail of Tire-Wear Particle Leachate** [111].

In this study, tyre wear particle leachates were used to assess their toxicity in euryhaline rotifer *B. plicatilis*. The tyre wear particle samples were created in a laboratory on a tyre simulator (drum).

The results showed that while acute toxicity was observed, and metals and PAHs were detected in the leachates, the levels remained under the threshold. The lethal concentration for 50% of the test subjects (LC50) was measured at 0.601 g/L for acute toxicity, and when testing chronic toxicity, reproduction and hence population growth were significantly decreased. The tyre leachates were able to induce oxidative stress, as the levels of ROS (reactive oxygen species) and MDA (malondialdehyde) were significantly increased after exposure. A significant increase in the activity of the antioxidant enzymes SOD, catalase (CAT), glutathione peroxidase (GPx), and glutathione S-transferase (GST) was also observed.

The gene analysis showed that exposure to leachates induced a transcriptional dysfunction of 1082 differentially expressed genes (DEGs), with 532 up-regulated and 549 down-regulated genes. Out of the gene ontology (GO) terms that were down-regulated, most of them are related to cellular metabolic and biological processes. The up-regulated DEGs were mostly related to endocytosis, the spliceosome, and the ribosome pathway, while the down-regulated ones were related to HIF-1 signalling pathways, fatty acid degradation, and ABC (ATP binding cassette) transporters. The toxicity pathway analysis showed cardiac hypertrophy, cellular homeostasis, energy metabolism, cell death, and cellular dysfunction as the main toxicity pathways.

**2022: Toxicity of Tire Rubber Microplastics to Freshwater Sediment Organisms** [74].

In this study, two freshwater sediment dwellers were used to assess the toxicity of tyre rubber particles using different types of sediment and different sizes and concentrations of particles. The samples were obtained as a fine powder after cryo-grinding, which is not a representative method.

The results showed that the survival of *C. riparius* larvae was not affected by exposure to tyre particles at any concentration tested, and the length of the larvae did not depend on the concentration. The emergence tests showed a significant difference between the control and the exposed groups in cumulative percentage; however, the final emergence rates did not vary. The production of egg ropes did not differ after exposure, except from in the highest concentration group, where an increase in reproduction was observed.

In the *L. variegatus* experiments, there were no significant effects on growth, survival, or reproduction observed, except from a slight difference in growth between the two sediments used.

**2022: Tyre Particle Exposure Affects the Health of Two Key Estuarine Invertebrates** [112].

In this paper, the health effects of tyre particles and their leachates on two estuarine invertebrates were assessed. The tyre particles were created by sanding and grinding four used car tyres, which is not a representative method.

The results showed that *S. plana* consumed 25 times more particles than *H. diversicolor*, which could explain why its health was impacted at much lower concentrations. Particles appeared to aggregate in the intestines in *S. plana* but not in *H. diversicolor*. Reductions in feeding and burial rates were also observed for *S. plana* but not for *H. diversicolor*.

No significant difference in the tissue lipid or carbohydrate concentration was observed for either organism. While the decrease in the protein concentration in *S. plana* was not dose-dependent, for *H. diversicolor*, the effect was dose-dependent. The total energy content was decreased for both species, probably caused by the decrease in protein concentration. At the highest concentrations, there was a dose-dependent increase in total glutathione in the tissues of *H. diversicolor* and a decrease in lipid peroxidation in the digestive glands in *S. plana*. Lastly, there was no observed mortality for *H. diversicolor* and only a low mortality for *S. plana*.

**2022: Acute Toxicity of Tire Wear Particles, Leachates and Toxicity Identification Evaluation of Leachates to the Marine Copepod, *Tigriopus japonicas*** [113].

In this paper, the toxicity of tyre wear particles in *T. japonicus* was assessed in vivo. The particle samples used in this study were collected by a waste tyre crushing factory and consisted of a random selection of mostly large truck tyres ground into small particles.

The results from the 96 h test showed the LC50 to be 771.4 mg/L for particles and 5.34 g/L for leachates. Zn was identified as the main cause of the toxicity by the toxicity identification evaluation study. Furthermore, the organic compound benzothiazole exhibited an antagonistic effect with zinc.

**2023: Toxicity of 6PPD Quinone to Four Freshwater Invertebrate Species** [75].

In this paper, the toxicity of 6PPDQ was assessed in vivo for four invertebrate species (*Hexagenia* spp., *Daphnia magna*, *Planorbella pilsbryi*, and *Megalonaias nervosa*). The results show that for all four species, the highest concentrations tested did not result in significant mortality, indicating that freshwater invertebrates are probably not as sensitive to 6PPDQ as some salmonid species.

**2023: Toxicological Effects of Tire Rubber-Derived 6PPD Quinone, a Species-Specific Toxicant, and Dithiobisbenzanilide (DTBBA) in the Marine Rotifer *Brachionus koreanus*** [114].

In this study, the toxicity of 6PPDQ and DTBBA was assessed in *Brachionus koreanus*. The results showed only moderate toxicity for 6PPDQ; however, they showed a significant response in population growth and fecundity for DTBBA at higher concentrations. The varying toxicity between the two compounds could be attributed to the significant increase in reactive oxygen species for DTBBA.

**2024: Aging Increases the Particulate- and Leachate-Induced Toxicity of Tire Wear Particles to Microalgae** [78].

In this paper, the toxicity of aged tyre wear particles (TWPs) and leachates was assessed in vivo through experiments on microalgae. The particle samples were collected by grating a used tyre with a stainless steel grater, which is not a representative method. In order to create aged particles, the virgin tyre wear samples were exposed to UV irradiation and two different doses of K_2_S_2_O_8_ to simulate short- and long-term aging.

The results showed that aged particles exhibited enhanced toxicity towards microalgae, compared to virgin particles, because of their increased leaching potential and physiochemical damage. The growth of microalgae was inhibited by all TWPs tested, at all concentrations on the first day of exposure. The contents of both chlorophyll a (Chla) and carotenoids (Car) increased over time, although Chla was more sensitive to the exposure. There were no significant dose-dependent effects of TWPs observed on the SOD and MDA contents; however, there were significant differences between leachates derived from virgin and aged particles. The findings show that exposure to any TWPs and their leachates altered the metabolic profiles, and especially UV- and high-dose-K_2_S_2_O_8_-aged particles had the biggest effect. Lastly, the results showed more significantly changed metabolites in the particle treatments compared to the leachates, indicating that the toxic effects of whole particles were greater than those of leachates.

**2024: A Ubiquitous Tire Rubber Additive Induced Serious Eye Injury in Zebrafish (*Danio rerio*)** [115].

In this paper, the toxicity of tyre additives was assessed in vivo in zebrafish. The chemicals used as tyre additives in order to simulate a tyre leachate were purchased. The chemicals used were aniline, benzothiazole (BT), 2-mercaptobenzothiazole (MBT), 1, 3-diphenylguanidine (DPG), and methimazole (MMI).

The results showed that MBT, BT, DPG, DTG, and aniline were highly suspected compounds with thyroid peroxidase (TPO) enzyme inhibition potency. No significant changes were observed in the eye structure for MMI, DPG, aniline, BT, or MBT. No significant decrease in eye size was found for DPG, BT, and aniline; however, both MBT and MMI exposure led to a significant decrease. However, when MBT was absent, the effect was not observed, which indicates that the main compound that causes serious eye development inhibition is MBT. Exposure to MBT also caused a significant decrease in cell density.

Many genes were up- or down-regulated after exposure to MBT or MMI. The authors explain that alterations in TPO expression seem to influence the expression of opsin-related genes, disrupting the thyroid system and the opsin protein synthesis pathways. Out of the suspected compounds tested, only MBT exposure was able to cause a significant response, which means it is probably one of the main toxic compounds in tyre wear leachates.

**2024: Combined Toxicity of Pristine (p-) or Artificially Aged (a-) Tire Wear Particles (TWP) and Bisphenols to *Tigriopus japonicus*** [116].

In this study, the combined toxicity of both pristine and aged TWPs, as well as four bisphenols (bisphenol A (BPA), bisphenol F (BPF), bisphenol S (BPS), and bisphenol AF (BPAF)) was assessed in *Tigriopus japonicus*.

The tyre particle samples were collected from a tyre waste factory, and they were produced by grinding and milling end-of-life tyres. In order to age the particles, they were placed in artificial sea water under UV irradiation for 12, 24, and 36 days.

The results showed that TWPs were able to increase the toxicity of BPA and BPF and decrease the toxicity of BPAF. Surprisingly, for BPS, there was a synergistic toxic effect in the presence of p-TWPs, but a slightly antagonistic effect was observed in the presence of a-TWPs. a-TWPs were found to release more Zn than p-TWPs which can have a toxic effect on *T. japonicus*. In fact, the presence of Zn increased the toxicity of BPA and BPF, which is consistent with the toxicity results. At higher concentrations, BPA, BPF, and BPS were able to significantly increase the aggregation size of p-TWPs and a-TWPs. The aggregation formed by TWPs of certain sizes (90–110 μm) could also cause intestinal damage and lipid peroxidation.

**2024: Cocktail Effects of Tire Wear Particles Leachates on Diverse Biological Models: A Multilevel Analysis** [117].

In this paper, the aquatic toxicity of TWP leachates was assessed through in vivo (*R. salina* and *D. rerio*) and in vitro (U-2 OS cells and CALUX assay) testing.

The particle samples were created by milling and micronising an unused tyre. The chemical analyses revealed the presence of polyaromatic hydrocarbons and 4-tert-octylphenol in the leachates. The authors suggest that the main cause of the toxicity is the water-leachable organic compounds released from the particles.

Exposure to the leachates (1.5 to 1000 mg peq L^−1^) was able to inhibit algal growth and induce embryotoxicity, pigment alterations, and behavioural changes in *D. rerio*. Cell painting showed pro-apoptotic changes, while mechanism-specific gene-reporter assays showed endocrine-disrupting potential, particularly antiandrogenic effects.

**2024: Accumulation and Depuration of Tire Wear Particles in Zebrafish (*Danio rerio*) and Toxic Effects on Gill, Liver, and Gut** [118].

In this in vivo study, the accumulation and depuration of TWPs in zebrafish at three different concentrations was investigated, as well as the toxic effects on the gills, liver, and gut. The particle samples were created by grinding mini-car tyre scraps from repair shops in China.

The results showed that the particles could accumulate in the gills and gut for a long time, in a dose-dependent manner. TWPs also induced oxidative stress in the gills and liver. The liver showed a tendency to up-regulate metabolic processes at higher concentrations and down-regulate at lower particle concentrations. The high-concentration treatment significantly increased xenobiotic biodegradation and metabolism and lipid metabolism-related pathways, whereas the low-concentration treatment distinctly altered amino acid metabolism-related pathways.

#### Appendix A.2.3. Terrestrial

**2011: Effects of Crumb Rubber Waste as a Soil Conditioner on the Nematode Assemblage in a Turfgrass Soil** [119].

In this paper, the toxicological effects of crumb rubber on soil nematodes were assessed. After exposure, the plant parasites and omnivorous nematode population decreased, and the predatory nematode population increased. Soil bulk density and pH were decreased, and soil moisture was increased.

**2018: Exposure to Aged Crumb Rubber Reduces Survival Time During a Stress Test in Earthworms (*Eisenia fetida*)** [120].

In this study, earthworms were exposed to aged crumb rubber in order to assess its toxicity and compare it to new crumb rubber. Exposure did not reduce earthworm body weight, but it reduced survival time during a stress test. Microbial respiration rates were not impacted. High levels of zinc were present in the samples.

**2020: Dysbiosis in the Gut Microbiota of Soil Fauna Explains the Toxicity of Tire Tread Particles** [121].

In this paper, the toxicity of tyre tread particles was assessed by exposing soil worms to these particles and analysing their gut microbiota. Exposure caused a decrease in survival and reproduction and disrupted the microbiota of the worm gut and the soil. There was also an enrichment of microbial genera associated with opportunistic pathogenesis in the worm guts.

**2021: Ecotoxicological Effects of Micronized Car Tire Wear Particles and their Heavy Metals on the Earthworm (*Eisenia fetida*) in Soil** [122].

In this in vivo study, microplastics, including tyre particles, were used to assess their toxicity in an earthworm species. *E. fetida* was exposed to certain microplastics in an artificial soil at different concentrations and exposure times (14 or 28 days) in order to assess the bioaccumulation of heavy metals and the oxidative stress induced. The tyre samples used in this study were created by cryo-milling used tyre treads.

The earthworms ingested the particles, with a preference for smaller particles. The results showed that at higher concentrations there was a significant increase in d catalase and peroxidase activity and lipid peroxidation levels, as well as reduced activity of superoxide dismutase and glutathione S-transferase, indicating oxidative stress. There was also absorption of heavy metals dependent on the shape, size, and concentration of the particles. Lastly, the enzyme activity was significantly altered after exposure.

**2021: Exploring the Impacts of Microplastics and Associated Chemicals in the Terrestrial Environment—Exposure of Soil Invertebrates to Tire Particles** [123].

In this study, the toxicological impact of tyre wear particles was assessed by exposing three species to spiked soil and food. The tyre samples were created in the laboratory by cryo-grinding a mix of used tyres.

In soil, at high concentrations, there was a significant decrease in *F. candida* reproduction (38%) and survival (24%) and acetyl-cholinesterase (AChE) activity of *P. scaber* (65%). There was a non-dose-dependent slight decrease in the reproduction of *E. crypticus*. In food, at high concentrations, *F. candida* survival was also significantly reduced (38%).

**2021: Exposure to Heavy Metal and Antibiotic Enriches Antibiotic Resistant Genes on the Tire Particles in Soil** [124].

In this paper, the effects of tyre particles were assessed by incubating tyre particles in soil with added stress from heavy metals and/or antibiotics and analysing the bacterial community and the antibiotic-resistant genes (ARGs). Different ARGs were found in the samples spiked with tyre particles compared to the control, as well as a lower diversity in the bacterial community and significantly increased amounts of ARGs when placed under stress from heavy metals and antibiotics.

**2021: The Influence of Microplastics from Ground Tyres on the Acute, Subchronical Toxicity and Microbial Respiration of Soil** [125].

In this study, the phytotoxicity of tyre wear particles was assessed through a series of experiments on two different plant species (*Sinapis alba* L. and *Lepidium sativum* L.). There is no information about how the tyre wear samples were collected. Soils with 50 and 75% tyre particles showed decreased biological activity and CO_2_-C emissions. The results also showed subchronical phytotoxicity and a lower germination index compared to the control.

**2021: Time-Dependent Toxicity of Tire Particles on Soil Nematodes** [126].

In this paper, the time-dependent toxicity of tyre wear particles was assessed in *C. elegans* by incubating the soil for 30 or 75 days and through both short- and long-term exposure. The tyre wear samples were created from a used tyre with a belt grinding machine. The results showed that the incubation increased the toxicity of the tyre wear particles, and that the lifetime of the organisms was reduced faster in the treated groups, while the effective concentrations where much lower in the lifetime exposure compared to the short-term exposure.

**2022: Time-Dependent Immune Response in Porcellio Scaber Following Exposure to Microplastics and Natural Particles** [127].

In this paper, the time-dependent toxicity of tyre particles was assessed in a terrestrial crustacean and compared to the effect of polyester fibres, wood dust, and silica powder. The crumb rubber samples were created by cryo-milling used car tyre scraps. The results showed that after 4 days of tyre particle exposure the total number of haemocytes was decreased significantly, and the proportions of different haemocytes were altered. Furthermore, after 7 days of exposure, there was an increase in superoxide dismutase activity and metabolic activity. Survival and feeding were not altered.

**2022: Toxicity Assessment of Tire Particles Released from Personal Mobilities (Bicycles, Cars, and Electric Scooters) on Soil Organisms** [128].

In this study, the toxicity of tyre wear particles from different personal mobility vehicles was assessed by exposing a plant species and a springtail. The particle samples were created by grating new tyre treads collected from three different personal mobility vehicles (car, bicycle, and electric scooter). While bicycles and scooters changed the soil’s bulk density and water holding capacity and reduced plant growth, car tyre particles leached organic compounds and had severe effects on springtails.

**2022: Two Types of Microplastics (Polystyrene-HBCD and car Tire Abrasion) Affect Oxidative Stress-Related Biomarkers in Earthworm *Eisenia andrei* in a Time-Dependent Manner** [129].

In this study, earthworms were exposed to car tyre and polystyrene microplastics in natural soil for different exposure times in order to assess their toxicity. The tyre samples were obtained from a bulk product of recycled tyres.

The results showed that multiple biomarkers showed significant changes in activity; however, the recovery of most enzymatic activities could be observed after 28 days. Only minor effects could be observed on a subcellular level at environmentally relevant concentrations.

**2023: Modulation of Chlorpyrifos Toxicity to Soil Arthropods by Simultaneous Exposure to Polyester Microfibers or Tire Particle Microplastics** [130].

In this study, the toxicity of tyre wear particles and polyester fibres was assessed through exposing soil arthropods. The tyre wear particles were created in a laboratory by cryo-milling used tyres. Exposure to tyre particles was able to reduce the lethality of chlorpyrifos and its effects.

**2023: Toxic and Biodegradation Potential of Waste Tires for Microorganisms Based on Two Experimental Designs** [131].

In this study, the toxicity of tyre rubber and tyre leachates was assessed in some unspecified soil microorganisms. Two tests were run, one with the leachates from the tyres and another one with the solution containing the tyre scraps. The rubber samples were created by grinding a mixture of tyres from personal transport.

When testing the consumption of dissolved oxygen in the assay over 28 days, the results indicated no biodegradation in any sample; however, the toxicity was higher when the microorganisms were exposed to the solution with tyre shred particles. The methyl tetrazolium test (MTT) viability assay showed 28% inhibition of the viability of microorganisms in samples with tyre particles, compared to 9% inhibition in the leachates.

**2023: Exposure to 6-PPD Quinone at Environmentally Relevant Concentrations Causes Abnormal Locomotion Behaviors and Neurodegeneration in *Caenorhabditis elegans*** [132].

In this study, the neurotoxicity of 6-PPDQ was assessed in nematode worms in vivo by exposing *Caenorhabditis elegans* to environmentally relevant concentrations.

The results showed that 0.1–10 μg/L of 6-PPDQ caused several forms of abnormal locomotion behaviours, as well as the neurodegeneration of D-type motor neurons at 10 μg/L. This was associated with the activation of the Ca^2+^ channel DEG-3-mediated signalling cascade in which many gene expressions changed.

**Table A1:** (**a**) Studies investigating the impact of brake particles on human/mammalian tissues. (**b**) Studies investigating the impact of brake particles on aquatic species.

**(a)**						
**Year**	**Ref.**	**Source**	**Parameters Examined**		**Testing Conditions**	**Main Conclusions**
2009	[52]	Prototype SM brake pad material emulating LM European brake system.	Brake wear debris (non-airborne) and ball-milled dust in vitro mutagenic potency and in vivo pulmonary toxicity via different types of assays.		Laboratory brake wear sampling (non-airborne dust) on a brake dyno in A04D wear test. Brake temperature up to 500 °C.	Brake wear debris proved toxic to *E. coli* strain after metabolic activation (Cu). No other significant biological effects were observed. Not clear if the prototype pads are representative of real-world commercial brakes.
2009	[49]	Brake system of one vehicle—does not specify what kind of vehicle or the brake type.	Brake wear particles and debris in vitro toxicological effects on epithelial lung cells.		Enclosed chamber with direct exposure of the cells to freshly generated brake dust. Two braking protocols were applied.	Increase in oxidative stress and pro-inflammatory response in lung cells was observed with fresh brake particles. No cytotoxicity or cell morphology deterioration.
2015	[50]	Different drum brakes from passenger cars. Mixed brake dust samples of light-duty brakes.	Brake wear debris and aged dust ability to generate reactive oxygen species (ROS).		Deposited dust on the drum brakes of various light-duty vehicles. Representative of urban driving and high-speed braking.	Aged brake wear particles are not extremely potent in forming ROS.
2015	[53]	Brake dust alone and in combination with added chrysotile and crocidolite asbestos.	Fate and pathological response in the lung and pleura of brake dust following short-term inhalation exposure in vivo.		Grinding of chrysotile-containing brake drums.	The crocidolite asbestos fibres were persistent through the lifetime and produced an inflammatory response in the lung, whereas the brake dust alone and brake dust with chrysotile samples were biosoluble and caused no significant pathological response.
2016	[47]	Prototype SM brake pad material emulating LM European brake system.	Brake airborne PM in vitro genotoxicity assay in human peripheral lymphocyte cells.		Laboratory brake wear sampling on a brake dyno with AK-Master. Brake temperatures up to 550 °C result in high particle number.	Exposure to brake particles has an impact on DNA damage of lymphocytes. Presence of crystalline metal is considered relevant. Not clear if the prototype pads are representative of real-world commercial brakes.
2016	[46]	Prototype SM brake pad material emulating LM European brake system—two commercial brake systems were also ball-milled.	Brake wear debris (non-airborne) in vitro toxicity and mutagenicity via different types of assays.		Laboratory brake wear sampling (non-airborne dust) on a brake dyno with A04D wear test. Brake temperature up to 500 °C.	The non-airborne debris of the model material contained compounds with toxic and mutagenic properties. Not clear if the prototype pads are representative of real-world commercial brakes.
2018	[54]	Chrysotile-containing brake dust and chrysotile or crocidolite asbestos.	Dose–response and fate in the lung and pleura of brake dust in a 28-day in vivo quantitative inhalation toxicology study.		Grinding of brake shoes.	Chrysotile samples caused a slight inflammatory response, but the fibres were able to be cleared by alveolar macrophage clearance. The crocidolite fibres were not able to be cleared, and they caused a significant inflammatory response and mesothelial pathology. The brake dust samples showed no significant pathological response.
2018	[87]	Different types of brake systems (different pads) used in passenger cars.	Brake PM and dust in vitro toxicity in human epithelial and primary immune cells.		Laboratory brake dust sampling on a brake dyno under urban driving conditions. High brake temperatures were observed up to 500 °C.	Airborne LM brakes do not induce any adverse biological effects in the in vitro lung multicellular model. Bigger particles from NAO brakes caused inflammatory responses and affected cell viability and morphology. Presence of TiO_2_ is considered relevant.
2018	[88]	Different types of brakes mounted on light-duty vehicles and tested on a brake dynamometer.	In vitro toxicity of brake wear debris, aged dust, and nanoparticles in the respiratory epithelium.		Deposited dust on the wheel parts of light-duty vehicles and a brake dynamometer.	Short-term loss of viability but with limited pro-inflammatory effects. Cytotoxicity of brake wear seems to be particle-size-independent. Substantial amount of nanoparticles with metallic content is considered relevant.
2019	[80]	Different brake systems (LM, SM, NAO, hybrid pads) and studded tyres.	Brake and tyre PM_2.5_ in vivo toxicity through exposure in mice.		Lab for both sources. PM_2.5_ sampling on a brake dyno. Unrealistic highway testing sequence—tyre wear particles on a road simulator under normal driving conditions (speed of 70 km/h).	No significant responses observed. No cytotoxicity or oxidative stress was observed. Different potency to induce inflammatory responses by the different types of brakes.
2020	[51]	Mixed brake dust samples from different types of drum brakes of heavy-duty vehicles.	Brake wear debris and aged dust in vitro toxicity in human airways through exposure to airway macrophages.		Deposited dust on the drum brakes of various heavy-duty vehicles. Representative of urban driving and high-speed braking.	Similar toxicological profiles of brake and diesel samples. Both trigger cytokine secretion, impair phagocytic capacity, and disrupt mitochondrial integrity. The metal content of brake dust is considered relevant.
2020	[59]	Four different brake pad/disc systems of light-duty vehicles.	Brake wear PM_2.5_ in vitro biological impacts.		Brake dynamometer under realistic driving/braking conditions.	PM_2.5_ with higher Cu induced cell toxicity that correlated with Cu concentration.
2021	[55]	Chrysotile-containing brake dust compared to TiO_2_, chrysotile, crocidolite, or amosite asbestos.	Dose–response and fate in the lung and pleura in a 90-day in vivo quantitative inhalation toxicology study.		Grinding of brake shoes.	Brake dust and chrysotile showed no significant pathological or tumourigenic response; however, crocidolite and amosite showed inflammation, microgranulomas, persistent fibrosis, and a dose-related lung tumour response.
2023	[48]	Different types of brake systems (different pads including LM, SM, and NAO pads).	In vitro cytotoxicity of brake wear PM_10_-bound PAHs and plasticisers in human alveolar epithelium cells.		Laboratory brake wear sampling on a brake dyno with one severe and two milder braking protocols. Brake temperature up to 550 °C over the severe cycle.	Brake PM from low-steel, high-steel, and non-asbestos organic brake pads decreases cell viability. No increase in intracellular ROS and no cell cycle arrest were observed.
2023	[89]	Different types of brakes mounted on light-duty vehicles and tested on a brake dynamometer.	In vitro toxicity of brake wear debris, aged dust, and nanoparticles in the respiratory epithelium.		Deposited dust on the wheel parts of light-duty vehicles and a brake dynamometer.	Brake wear nanoparticles do not cause overt cytotoxicity and inflammation. They can translocate through the epithelial barrier and increase mucus production. Indication of acute inflammation and potential for chronic obstructive pulmonary diseases.
2023	[58]	Different types of brake systems of 3 vehicles.	In vitro toxicity of unspecified brake dust sample of up to 50 μm in a bronchial epithelial cell line.		Brush deposited dust from the brake discs of 3 vehicles. Very high Cd concentrations indicate use of old brake linings.	Brake dust demonstrates cytotoxicity and a significant secretion of the pro-inflammatory cytokine IL-8. Negative effects were attributed to Cu and Cd. No oxidative stress was noted.
**(b)**						
**Year**	**Ref.**	**Source**	**Species**	**Parameters Examined**	**Testing Conditions**	**Main Conclusions**
2017	[81]	Brake system of three vehicles.	*Echinogammarus Veneris* (amphipod crustaceans).	In vivo assessment of the genotoxic and oxidative stress effects of brake wear debris on *Echinogammarus veneris*.	Collected from the brake linings of three different vehicles after use.	Brake dust was shown to cause less genotoxic damage than the natural dusts. Oxidative stress was demonstrated for brake dust.

**Table A2:** (**a**) Studies investigating the impact of tyre particles on human/mammalian tissues. (**b**) Studies investigating the impact of tyre particles on aquatic species. (**c**) Studies investigating the impact of tyre particles on terrestrial species.

**(a)**						
**Year**	**Ref.**	**Source**	**Parameters Examined**		**Testing Conditions**	**Main Conclusions**
2005	[90]	Tyre debris eluates and organic extracts.	Tyre debris toxicity in A549 human alveolar cells and HepG2 human liver cells in vitro and in *X. laevis* embryos in vivo.		Laboratory through cryo-fracturing of tyre scraps.	Dose-dependent increase in DNA damage and decreased cell proliferation. Morphological changes. Time-dependent increase in Zn in HepG2 cells. Increased mortality rate in *X. laevis* embryos and malformed larvae.
2005	[56]	Tyre debris organic extracts.	Tyre debris cytotoxicity in A549 human alveolar cells.		Laboratory by rotating a new tyre against a steel brush.	Dose- and time-dependent inhibitory effect on the reduction of MTT and dose-dependent increase in cell mortality and DNA strand breakage. Multiple structural alterations.
2006	[66]	Studded tyre wear PM_10_ across two different pavements compared to other sources.	Studded tyre wear particle inflammatory response in human macrophages and nasal and bronchial epithelial cells.		VTI road simulator using studded tyres and two types of pavements: dense asphalt with granite and stone mastic asphalt with quartzite.	Granite pavement caused a higher cytokine release compared to quartzite. Tyre particles caused a higher decrease in cell viability compared to street and subway particles. None of the samples caused a cytokine release from nasal epithelial cells.
2006	[82]	Tyre–road wear particles compared to other sources (studded and winter tyres).	Genotoxicity of studded tyre–road wear particles in A549 human alveolar cells.		VTI road simulator using 1 studded tyre on ABT pavement, 2 studded tyres on ABS pavement, and 1 friction (winter) tyre on sanded ABS pavement.	All particles were able to cause DNA damage; however, winter tyres on sanded ABS pavement caused significantly higher DNA damage. Studded tyres on ABT pavement caused an increase in IL-8 and TNF-α, and studded tyres on ABS pavement caused an increase in IL-6, IL-8, and TNF-α.
2007	[67]	Studded tyre wear PM_10_ across two different pavements compared to other sources.	Studded tyre wear particle inflammatory effects on mouse macrophage cells.		VTI road simulator using studded tyres and two types of pavements: dense asphalt with granite and stone mastic asphalt with quartzite.	All particles were able to induce cytokine release. Granite caused much higher release of TNF-α and IL-6 compared to quartzite. Both granite and quartzite induced similar lipid peroxidation and ROS formation, but only granite induced NO production.
2007	[63]	Tyre debris organic extracts.	Tyre debris cytotoxicity in A549 human alveolar cells.		Laboratory by rotating a new tyre against a steel brush.	Dose-dependent increase in % of Trypan Blue-positive cells. Time- and dose-dependent increase in LDH and lipid microdomains. No difference in cell proteins. Twenty-fold increase in HO-1 content in DRM. Increase in invaginations but no other morphological differences.
2008	[91]	Tyre debris organic extracts.	Tyre debris toxicity in A549 human alveolar cells (ROS production and Hsp 70 expression).		Laboratory through cryo-fracturing of a new tyre.	Significant dose-dependent ROS production. Higher Hsp70 expression with higher exposure times and lower doses.
2008	[68]	7 combinations of tyres, pavements, and traction materials compared to other sources.	Tyre wear particle toxicological effects on human monocytes.		VTI road simulator using studded tyres, non-studded winter tyres, ABT with granite, ABS with quartzite, no traction material, crushed stone as traction material, and natural sand as traction material.	All particles were able to induce a cytokine release. Granite caused much higher release of TNF-α, IL-8, and IL-6 compared to quartzite.
2008	[60]	Studded tyre–road wear particles compared to other sources.	Studded tyre–road wear particle genotoxicity through in vitro experiments on human lung epithelial cells.		VTI road simulator using studded tyres and ABT pavement.	Tyre–road samples caused the least amount of mitochondrial depolarisation (37%) and showed almost no increase in ROS.
2008	[43]	Tyre particles and metals (Zn and Cu).	Tyre particle cardiopulmonary toxicity in mice through intratracheal installations.		Laboratory by grinding fresh tyre material (recycled styrene butadiene rubber) and 2 tyre scrap samples.	There was a higher increase in total lavageable cells in the scrap sample. The Zn + Cu sample caused a much higher effect compared to the individual metals. All samples increased BALF lung injury markers and decreased mitochondrial aconitase activity.
2009	[44]	Tyre wear particles, PM_2.5_ and PM_10_.	(Size-fractioned) Tyre particle lung toxicity in mice (in vivo through intratracheal instillation).		Laboratory through cryo-fracturing of tyre scraps.	PM_2.5_ showed stronger cytotoxic effects, although PM_10_ did show some cytotoxicity at higher doses. PM_2.5_ was more dispersed in alveolar spaces and was not able to be cleared out.
2010	[45]	Tyre wear particles, PM_2.5_ and PM_10_, compared to other sources.	(Size-fractioned) Tyre particle lung toxicity in mice (in vivo through intratracheal instillation).		Laboratory through cryo-fracturing of tyre scraps.	TP_2.5_ reached the alveolar spaces and caused an acute inflammatory response, whereas TP_10_ reached bronchial spaces and did not cause a significant response.
2011	[92]	Studded tyre wear, PM_10_.	Tyre wear particles’ toxicology through protein release from human macrophages after TWP and ETX exposure.		VTI road simulator using studded tyres and dense asphalt pavement with granite.	TWP exposure: 7 down-regulated and 3 up-regulated proteins. Proteins involved in inflammatory response were up-regulated, and proteins involved in cellular functions were down-regulated. ETX exposure: 4 down-regulated and 2 up-regulated.
2012	[57]	Tyre and road wear particles.	Tyre wear particle lung toxicology through in vivo inhalation in rats.		Laboratory drum testing system with asphalt pavement using 1 summer silica tyre, 1 winter silica tyre, 1 carbon-black tyre.	No general toxicity, cytotoxicity, or inflammatory potential was observed. A few focal areas of subacute inflammatory cell infiltration were observed at higher doses.
2019	[93]	Truck and passenger tyre wear particles.	Tyre wear particles’ genotoxicity in mice macrophage RAW264.7 cells.		Laboratory through cryo-fracturing of a passenger tyre and a truck tyre.	Both samples caused a reduction in cell viability, decrease in metabolic activity, and increase in TNF-α. Truck tyres overall caused less toxicity than passenger tyres.
2020	[61]	Microplastics from polymer pellets and truck tyre materials.	Polymer and tyre microplastic ingestion toxicity in vitro through a 3D intestinal model.		Laboratory through cryo-fracturing of polymer pallets and tyre materials.	No significant cytotoxicity or release of pro-inflammatory cytokines was observed. There was no change in the barrier integrity of the co-cultures.
2022	[94]	Photoaged tyre wear particles (environmentally persistent free radicals).	Photoaged tyre wear particles’ toxicology through in vitro experiments on mice macrophages.		Laboratory through cryo-fracturing of tyre scraps.	TWPs caused a decrease in cell viability and an increase in ROS generation and mRNA levels for inflammatory factors. The toxicological effects were stronger with higher irradiation time suggesting that free radicals play a role.
2022	[62]	Road dust and tyre wear particles’ organic extract (PAHs).	Road dust mutagenicity with bacterial assays (Ames test).		VTI road simulator using 2 different summer tyres and with in situ resuspension chambers in 2 cities.	No mutagenic response was observed, although the testing conditions were not satisfactory, so the results are not reliable.
2022	[95]	Tyre wear microplastic particles.	Tyre wear particle lung toxicology through in vivo inhalation in mice.		Laboratory through cryo-fracturing of a tyre.	TWP exposure caused restricted ventilatory dysfunction and fibrotic pathological response in mice. Major histopathological changes were observed in the lungs consistent with pulmonary fibrosis. Cytoskeleton rearrangement and cell migration were observed in vitro.
2023	[69]	Tyre wear particles.	Tyre wear particle lung toxicity through a 3D in vitro model of human airway organoids.		Laboratory through cryo-fracturing of a tyre.	TWPs induced significant cytotoxicity and ROS generation and increased cytokines such as TNF-α and IL-6. Particles were found inside the cells and clustered around the cells. The exposed hAOs decreased in size. TWPs caused an increase in early and late apoptotic cells.
2023	[64]	Tyre wear particles.	Tyre wear particles’ toxicity in mice macrophage RAW264.7 cells.		Laboratory on-road simulator and then cryo-milled.	There was no sign of DNA damage, and toxicological responses were only observed after prolonged stress and high concentrations in the proliferation study, the gene expressions, and the immunoblot analysis.
2023	[70]	6PPD and 6PPDQ.	6PPD and 6PPDQ hepatotoxicity in mice in vivo.		-	Both substances were found to bioaccumulate in the liver in a dose-dependent manner. Higher doses increased the liver’s weight and the triglyceride levels. The hepatic metabolism and immune response were altered.
2023	[71]	6PPDQ.	6PPDQ toxicology in vivo in mice through intraperitoneal injections.		-	In the single-injected mice, there were no significant changes in organ indexes or biochemical parameters, and no pathological changes were observed. Repeated injections caused many changes in organ indexes. Significant pathological changes occurred in the organs. The biochemical parameters for the liver and kidney were significantly up-regulated, and there was 6PPDQ accumulation in the liver and lungs.
2024	[96]	Combined exhaust gases, benzo[a]pyrene, and tyre particles.	Combined emissions’ toxicity in mice cell macrophages in vitro.		Laboratory by cryo-grinding commercial tyres.	No cytotoxicity or ROS production. B[a]P caused an inflammatory response, and tyre particles caused a size-dependent response; exhaust gases did not. For the larger particles, there was a disproportionate increase in TNF-α production, suggesting a synergistic effect.
2024	[65]	Ground tyre particles and actual tyre and road wear particles.	In vitro toxicity of ground TP and actual TRWP emissions in mice cells.		TPs: cryogenic grinding of a tyre. TRWPs: on-road sample collection from a vehicle.	No cytotoxicity or oxidative stress. Both particles induced a concentration-dependent proinflammatory response, but the TNF-α production was slightly higher for TRWPs.
**(b)**						
**Year**	**Ref.**	**Source**	**Species**	**Parameters Examined**	**Testing Conditions**	**Main Conclusions**
2005	[56]	Tyre debris leachates.	*R. subcapitata* (microalga), *D. magna* (crustacean), and *X. laevis* (frog, amphibian).	Tyre debris leachates’ toxicity in vivo and in vitro (FETAX) in aquatic organisms.	Laboratory by rotating a new tyre against a steel brush.	The results showed strong dose-dependent growth inhibition in *R. subcapitata* and death rate in *D. manga* and *X. laevis*.
2007	[97]	Tyre debris organic extracts.	Xenopus (frog).	Tyre debris organic extracts’ embryotoxicity in frogs (FETAX test).	Laboratory by cryo-fracturing tyre scraps.	The NOEC was at 50 mg/L, mortality and percentage of malformed larvae was increased at 80 mg/L, and the probit analysis gave a 144.6 mg/L TC50. There were mostly ocular malformations and severe vacuolisation and necrosis in the liver and axial musculature.
2009	[98]	Tyre debris (Zn^2+^) and ZnCl_2_.	*R. sylvatica* (frog).	Tyre debris (Zn) toxicology in frog larvae (effects on metamorphosis).	Laboratory from used tyre scraps.	Both the exposures caused an increase in the time for larvae to complete metamorphosis. The increase metamorphosis time caused a decrease in the subject’s mass.
2009	[99]	Sequential tyre powder leachates.	*D. magna*, *C. dubia* (crustaceans), *P. subcapitata* (algae), and *D. rerio* eggs (fish).	Toxicity of tyre leachates in vivo and in vitro in aquatic life.	Laboratory by abrading tyres with a rasp and then leachating the powder (1 slightly worn and 2 heavily worn tyres).	The slightly worn tyre was shown to cause the most significant toxic response. The sequential leachates were also shown to be much less toxic than the first one. Toxicology was mostly attributed to zinc.
2010	[100]	Tyre particle leachates.	*U. lactuca* (marine macroalga).	In vivo toxicity to macroalgae and Zn release.	Abrasion of 20 end-of-life car tyres with stainless steel file.	Exposure to increasing concentrations of leachate resulted in a non-linear reduction in the efficiency of photochemical energy conversion and an increase in Zn accumulation.
2011	[101]	Tyre and road wear particle sediment elutriate and leachate.	*P. subcapitata* (algae), *D. manga* (crustacean), and *P. promelas* (fish).	In vivo acute toxicity of tyre and road particles to aquatic life.	Road simulator laboratory in an interior drum testing system containing actual asphalt pavement in cassettes (1 summer and 1 winter silica-based tyre, 1 carbon-black-based summer tyre).	No concentration response was observed, but some toxicity was observed at higher incubation temperatures.
2013	[102]	Tyre and road wear particle sediments and elutriates.	C. dilutus (holometabolous aquatic insect), *H. azteca*, *C. dubia* (crustaceans), and *Pimephales promelas* (fish).	Chronic toxicity of tyre and road wear particles in vivo in water- and sediment-dwelling organisms.	Road simulator laboratory in an interior drum testing system containing actual asphalt pavement in cassettes (1 summer, 1 winter silica-based tyre, 1 carbon-black-based summer tyre).	The sediments caused a mild growth inhibition in C. dilutes but had no effect on growth or reproduction in *H. azteca*. The elutriates caused a slight diminished survival in larval *P. promelas* but had no effect on growth or reproduction in *C. dubia*.
2018	[73]	Tyre particles.	*G. pulex*, *A. aquaticus* (crustaceans), *L. variegatus*, and Tubifex spp. (worms).	Tyre tread particle toxicity in freshwater invertebrates.	Laboratory through cryo-grinding of a tyre tread.	The results showed no effect on the survival, growth, and feeding rate of *G. pulex* and *A. aquaticus*, the survival and growth of *Tubifex* spp., and the number of worms and growth of *L. variegatus*.
2019	[103]	Tyre wear particles and tyre leachates.	*H. azteca* (crustacean).	Acute and long-term toxicology of tyre wear particles and leachates in freshwater organisms.	Laboratory by surface abrasion of one road-worn tyre for tyre wear particles; TWPs were used for leaching.	Mortality, reproductive output, and net growth were significantly affected at higher concentrations. Tyre wear particles showed a typical concentration–response pattern, while leachates did not.
2020	[83]	Tyre rubber leachates compared to other plastics.	*R. subcapitata*, *S. costatum* (microalgae), and *M. galloprovincialis* (mussel).	Tyre wear toxicity in aquatic life (freshwater and marine).	Laboratory using tyre-derived granulate reference materials.	Tyre leachates were found to be amongst the most toxic samples tested. They caused the inhibition of algal growth, significant down-regulation of gamete fertilisation, and inhibition of normal embryonic development in mussels.
2020	[77]	Tyre particle leachates.	*P. promelas* (fish).	Tyre leachates’ in vivo toxicity in fish comparing different physical stressors (temp., UV, CO_2_, mechanical stress).	Laboratory by manually cutting small pieces from new tyres.	The results showed that the variations in temperature and mechanical stress caused a significant change in toxicity, whereas UV and CO_2_ exposure caused milder effects.
2020	[104]	Tyre particles (crumb rubber).	*F. heteroclitus* and *P. promelas* (fish).	In vivo toxicity through 7-day static renewal exposure.	Crumb rubber from passenger and truck tyres.	Highest concentration tested caused partial mortality in *P. promelas*. Bile fluorescence and EROD assay indicated that there was a presence of PAHs.
2021	[105]	Pristine tyre particles and leachates compared to road-worn TWPs and leachates.	*H. azteca* (crustacean).	Tyre wear particle suspensions’ and leachates’ toxicity in aquatic life.	Laboratory by grinding two tyres (new and used).	New TP suspensions were more toxic than those of worn TPs; however, leachates were equally toxic. New TP suspensions showed no significant effects on mortality and reproduction, but growth was significantly reduced at the highest concentration tested.
2021	[76]	Tyre wear particle leachates.	Coho and *Chum salmon* (fish).	Tyre leachates’ in vivo toxicity in coho and chum salmon.	Laboratory by grinding nine unique tyres (2 new, 7 used).	Coho salmon displayed significant behavioural changes, mortality, and changes in blood parameters after exposure. Chum salmon was unaffected by exposure.
2021	[84]	Tyre wear particles compared to polystyrene microplastics.	*C. riparius* (midge).	Tyre wear particles’ in vivo toxicity in a midge.	Laboratory through cryo-grinding of an end-of-life tyre.	Several genes encoding heat shock proteins were overexpressed, and genes coding for manganese superoxide dismutase and for the FK506-binding protein were altered. Microplastics caused cellular stress which led to some gene alterations but did not cause any mortality.
2022	[85]	Tyre wear particles compared to PET particles (leachates).	Freshwater bacterial community.	Tyre wear microbiological effects on freshwater ecosystems.	Laboratory by shredding of a used tyre.	TWPs caused increased bacterial growth and the creation of a biofilm that was much less diverse compared to PET.
2022	[106]	Tyre wear particles and leachates.	*A. bahia* (crustacean) and *M. beryllina* (fish).	Tyre particles’ and leachates’ toxicity and behavioural changes in aquatic organisms.	Laboratory through cryo-milling of a tyre tread.	TWPs caused significant alterations in swimming behaviours, a concentration-dependent reduction in growth after exposure to microparticles for both species, and a reduction in growth after exposure to nanoparticles for *M. beryllina*.
2022	[107]	Tyre particles and leachates.	*D. rerio* (fish) and *D. manga* (crustacean).	Toxicity of micro and nano tyre particles and leachates in freshwater organisms.	Laboratory through cryo-milling of a tyre.	Nano tyre particles and leachates caused an increased mortality and sublethal malformations, but micro tyre particles did not.
2022	[108]	6PPD and 6PPDQ.	*D. rerio* (fish).	6PPD and 6PPDQ toxicity in fish larvae.	-	6PPDQ was found to be toxic, but no significant mortality was observed. There was a dose-dependent reduction in swimming performance in both compounds.
2022	[109]	Tyre wear (crumb rubber) leachates.	*F. heteroclitus* (fish).	Tyre leachates’ in vivo toxicity in fish with episodic exposure.	Crumb rubber.	CYP1A was up-regulated in gills, intestine, and liver, and there was a dose-dependent increase in bile fluorescence. There was increased 8-OHdG and total GSH and decreased MDA production.
2022	[110]	End-of-life tyre-derived granules and powder.	*P. subcapitata* (algae), *D. manga* (crustacean), and *D. rerio* (fish).	Tyre granules’ and powder suspensions’ acute and chronic in vivo toxicity in freshwater organisms.	ELT granules and powder from a tyre recycling plant.	Acute tests showed EC50 of over 100 μg/L for *D. magna* and *D. rerio*. Chronic tests showed an LOEC of 9.8 μg/L for *D. magna* and 10 μg/L for *D. rerio*. There were significant effects on the cell density of *P. subcapitata* at 100 μg/L.
2022	[86]	Tyre particles compared to polyester fibres.	*D. manga* (crustacean), *H. azteca*, *A. aquaticus* (crustaceans), and *L. variegatus* (worm).	Tyre particles’ acute and chronic in vivo toxicity in freshwater invertebrates.	End-of-life tyre granules collected from manufacturer and milled.	There was no effect on mobility after acute exposure, but there was an effect on survival and reproduction after chronic exposure in *D. magna*. There was no effect on survival for the other species; however, there was significant ingestion.
2022	[111]	Tyre wear particle leachates.	*B. plicatilis* (rotifer).	Tyre wear particle leachate in vivo toxicity in a euryhaline rotifer.	Laboratory from a tyre simulator (drum).	Acute toxicity was observed. Reproduction and population growth were decreased, and oxidative stress was induced after exposure. There was an increase in antioxidant enzymes and an induction of transcriptional dysfunction of 1082 DEGs.
2022	[74]	Tyre wear particles (microrubber).	*C. riparius* (midge) and *L. variegatus* (worm).	Tyre wear particle in vivo toxicity in freshwater sediment organisms.	Laboratory through cryo-grinding of an end-of-life tyre.	The results showed no significant effects on growth, survival, or reproduction.
2022	[112]	Tyre wear particles.	*S. plana* (mollusc) and *H. diversicolor* (worm).	Tyre wear particle in vivo toxicity in estuarine invertebrates.	Laboratory through grinding of used tyres (with liquid nitrogen).	*S. plana* had reductions in burial and feeding rates. Protein concentration and total energy content were affected. Total glutathione in *H. diversicolor* and lipid peroxidation in *S. plana* were changed.
2022	[113]	Tyre particles and leachates.	*T. japonicus* (crustacean).	In vivo toxicity to marine copepod.	Waste tyre crushing factory from heavy-duty trucks.	LC50 was 771.4 mg/L for particles and 5.34 g/L for leachates. Zn was identified as the main cause of the toxicity.
2023	[75]	6PPDQ.	*Hexagenia* spp. (insect), *D. magna* (crustacean), *Planorbella pilsbryi* (mollusc), and *M. nervosa* (mussel).	6PPDQ toxicity in vivo for four freshwater invertebrate species.	-	For all four species, the highest concentrations tested did not result in significant mortality.
2023	[114]	6PPDQ and DTBBA.	*Brachionus koreanus* (marine rotifer).	6PPDQ and DTBBA toxicity in a marine rotifer.	-	Only moderate toxicity was observed for 6PPQ; however, there was a significant response in population growth and fecundity for DTBBA.
2024	[78]	Aged tyre wear particles and leachates.	*P. tricornutum* (microalgae).	Aged TWPs’ and leachates’ in vivo toxicity in microalgae.	Laboratory by grating a used tyre.	Growth of microalgae was inhibited. Chla and Car increased over time. No significant effect on SOD and MDA. Overall, whole particles were more toxic than leachates and aged particles more than virgin ones.
2024	[115]	Tyre additives (leachates).	*D. rerio* (fish).	Tyre additives’ in vivo toxicity in fish (ophthalmological effects).	-	MBT (2-mercaptobenzothiazole) was found to be one of the main toxic compounds, as it caused a significant decrease in eye size and cell density. Many gene expressions were also changed.
2024	[116]	Pristine and aged TWPs and four bisphenols.	*T. japonicus* (crustacean).	In vivo toxicity of combined pristine and aged TWPs and four bisphenols in a crustacean.	Laboratory by grinding end-of-life tyres.	TWPs increased the toxicity of BPA and BPF and decreased the toxicity of BPAF. For BPS, there was a synergistic toxic effect with p-TWPs but a slightly antagonistic effect with a-TWPs.
2024	[117]	TWP leachates.	*R. salina* (microalga) and *D. rerio* (zebrafish) and U-2 OS cells and CALUX assay.	In vivo (*R. salina* and *D. rerio*) and in vitro (U-2 OS cells and CALUX assay) toxicity of TWP leachates.	Laboratory by milling unused tyres.	Leachates inhibited algal growth and induced embryotoxicity, pigment alterations, and behavioural changes in *D. rerio*. In vitro testing showed pro-apoptotic changes and endocrine-disrupting potential.
2024	[118]	Tyre wear particles.	*D. rerio* (zebrafish).	In vivo toxicity in the gills, liver, and gut and accumulation and depuration of TWPs.	Laboratory by grinding mini-car tyre scraps.	There was a dose-dependent TWP accumulation in the gills and gut for a long time, and oxidative stress was induced in the gills and liver. There were dose-dependent up- and down-regulations of metabolic processes by the liver.
**(c)**						
**Year**	**Ref.**	**Source**	**Species**	**Parameters Examined**	**Testing Conditions**	**Main Conclusions**
2011	[119]	Crumb rubber.	13 soil nematodes (mainly Helicotylenchus) (worm).	Crumb rubber toxicity and effects on nematodes and soil.	-	After exposure, the plant parasite and omnivorous nematode population decreased, and the predatory nematode population increased. Soil bulk density and pH were decreased, and soil moisture was increased.
2018	[120]	Aged crumb rubber.	*E. fetida* (worm).	Aged crumb rubber toxicity to earthworms.	-	Exposure did not reduce earthworm body weight, but it reduced survival time during a stress test. Microbial respiration rates were not impacted.
2020	[121]	Tyre tread particles.	*E. crypticus* (worm).	Tyre tread particle effects on soil fauna and worm gut microbiota.	Laboratory by grating a used tyre.	Exposure caused a decrease in survival and reproduction and disrupted the microbiota of the worm gut and the soil.
2021	[122]	Tyre tread particles.	*E. fetida* (worm).	Tyre particles’ effect on earthworms (bioaccumulation of heavy metals and oxidative stress).	Laboratory by cryo-grinding a used tyre tread.	Higher concentrations caused oxidative stress, as well as increased catalase and peroxidase activity and lipid peroxidation levels, and reduced activity of SOD and GST.
2021	[123]	Tyre particles.	*E. crypticus* (worm), *F. candida* (springtail), and *P. scaber* (crustacean).	Tyre particles’ toxicology when spiked in soil or food.	Laboratory through cryo-milling end-of-life tyres.	In soil, at high concentrations, there was a decrease in *F. candida* reproduction and survival and the AChE activity of *P. scaber*. In food, at high concentrations, *F. candida* survival was reduced.
2021	[124]	Tyre particles.	Soil bacterial community.	Tyre particles’ effect on bacterial community when spiked into soil.	Laboratory by grating a used tyre.	Different ARGs were found in the samples spiked with tyre particles compared to the control, and the amounts were significantly increased when placed under stress from heavy metals and antibiotics.
2021	[125]	Tyre wear particles.	*Sinapis alba* L. and *Lepidium sativum* L. (plants).	Tyre particle phytotoxicity (biological activity, CO_2_, germination index).	-	Soils with 50 and 75% tyre particles showed decreased biological activity and CO_2_-C emissions. There was a subchronical phytotoxicity and a lower germination index compared to the control.
2021	[126]	Tyre wear particles.	*C. elegans* (worm).	Tyre particle time-dependent toxicity (soil pre-incubation and exposure time).	Laboratory by grinding a used tyre.	The incubation increased the toxicity of the tyre wear particles, and the lifetime of the organisms was reduced faster in the treated groups, especially with lifetime exposure.
2022	[127]	Tyre wear particles compared to polyester fibres and natural particles.	*P. scaber* (terrestrial crustacean).	Tyre particle time-dependent toxicity (immune system).	Laboratory by cryo-milling used tyre scraps.	After 4 days of exposure, the total number of haemocytes was decreased, and the proportions of different haemocytes were altered. After 7 days of exposure, there was an increase in superoxide dismutase activity and metabolic activity.
2022	[128]	Tyre wear particles from cars, bicycles, and e-scooters.	*V. radiata* (plant) and *F. candida* (springtail).	Tyre particle toxicity in soil organisms.	Laboratory by grating new tyre treads from 3 different personal mobility means.	Bicycles and scooters changed the soil’s bulk density and water holding capacity and reduced plant growth. Car tyre particles leached organic compounds and had severe effects on springtails.
2022	[129]	Microplastics (tyre particles and polystyrene).	*E. andrei* (worm).	Time-dependent in vivo toxicity in earthworms.	Bulk product of recycled tyres.	Only minor effects could be observed on a subcellular level at environmentally relevant concentrations.
2023	[130]	Tyre wear particles compared to polyester microfibres.	*F. candida* (springtail) and *P. scaber* (terrestrial crustacean).	Change in chlorpyrifos toxicity after exposure to tyre particles for soil arthropods.	Laboratory by cryo-milling used tyres.	The lethality of chlorpyrifos and its effects were reduced significantly after exposure.
2023	[131]	Tyre rubber and tyre leachates.	Unspecified soil microorganisms.	Toxicity in microorganisms.	Laboratory by grinding tyres.	No biodegradation in any sample, but the toxicity was higher when exposed to tyre shred particles. MTT test showed 28% inhibition of the viability for the samples with particles.
2023	[132]	6PPDQ.	*C. elegans* (worm).	In vivo toxicity of 6PPDQ in nematodes.	6PPDQ.	A total of 0.1–10 μg/L of 6-PPDQ caused several forms of abnormal locomotion behaviours and the neurodegeneration of D-type motor neurons at 10 μg/L.

**Table A3:** Summary of materials used in studies about health effects of brakes.

Ref.	Non-Asbestos Organic (NAO)	Low-Metallic	Semi-Metallic	Hybrid Vehicle	Prototype	Asbestos Containing	Heavy-Duty (All)	Not Specified
Mammalians	[48,80,87]	[46,48,80,87]	[48,80]	[80]	[46,47,52,59]	[53,54,55]	[51]	[49,50,58,59,88,89]
Aquatic								[81]

**Table A4:** Summary of materials used in studies about health effects of tyres on mammalians.

Ref.	Summer	Winter	All-Season	Winter Studded	Reference Material	6PPDQ and Extracts	Heavy-Duty	Not Specified
Mammalians	[57,62,94]	[57,68,82]	[94]	[60,66,67,68,82,92]	[43]	[70,71]	[61,93]	[43,44,45,56,63,64,65,69,90,91,93,95,96]
Aquatic	[101,102]	[101,102]			[83,104,109]	[75,108,114,115,116]	[104,113]	[56,73,74,76,77,78,84,85,86,97,98,99,100,103,105,106,107,110,111,112,116,117,118]
Terrestrial					[119,120,129]	[125,132]		[121,122,123,124,125,126,127,128,130,131]
